# Global associations of key populations with HIV-1 recombinants: a systematic review, global survey, and individual participant data meta-analysis

**DOI:** 10.3389/fpubh.2023.1153638

**Published:** 2023-07-27

**Authors:** Nkazi Nchinda, Ramyiadarsini Elangovan, Jason Yun, Leslie Dickson-Tetteh, Shona Kirtley, Joris Hemelaar, WHO-UNAIDS Network for HIV Isolation and Characterisation, Alash'le G. Abimiku

**Affiliations:** ^1^Nuffield Department of Population Health, Infectious Disease Epidemiology Unit, National Perinatal Epidemiology Unit, University of Oxford, Oxford, United Kingdom; ^2^Centre for Statistics in Medicine, Nuffield Department of Orthopaedics, Rheumatology and Musculoskeletal Sciences, University of Oxford, Botnar Research Centre, Oxford, United Kingdom

**Keywords:** HIV, key populations, recombinant, CRF, URF, molecular epidemiology

## Abstract

**Introduction:**

Global HIV infections due to HIV-1 recombinants are increasing and impede prevention and treatment efforts. Key populations suffer most new HIV infections, but their role in the spread of HIV-1 recombinants is unknown. We conducted a global analysis of the associations between key populations and HIV-1 recombinants.

**Methods:**

We searched PubMed, EMBASE, CINAHL, and Global Health for HIV-1 subtyping studies published from 1/1/1990 to 31/12/2015. Unpublished data was collected through a global survey. We included studies with HIV-1 subtyping data of key populations collected during 1990-2015. Key populations assessed were heterosexual people (HET), men who have sex with men (MSM), people who inject drugs (PWID), vertical transmissions (VERT), commercial sex workers (CSW), and transfusion-associated infections (BLOOD). Logistic regression was used to determine associations of key populations with HIV-1 recombinants. Subgroup analyses were performed for circulating recombinant forms (CRFs), unique recombinant forms (URFs), regions, and time periods.

**Results:**

Eight hundred and eighty five datasets including 77,284 participants from 83 countries were included. Globally, PWID were associated with the greatest odds of recombinants and CRFs (OR 2.6 [95% CI 2.46–2.74] and 2.99 [2.83–3.16]), compared to HET. CSW were associated with increased odds of recombinants and URFs (1.59 [1.44–1.75] and 3.61 [3.15–4.13]). VERT and BLOOD were associated with decreased odds of recombinants (0.58 [0.54–0.63] and 0.43 [0.33–0.56]). MSM were associated with increased odds of recombinants in 2010–2015 (1.43 [1.35–1.51]). Subgroup analyses supported our main findings.

**Discussion:**

As PWID, CSW, and MSM are associated with HIV-1 recombinants, increased preventative measures and HIV-1 molecular surveillance are crucial within these key populations.

**Systematic review registration:**

PROSPERO [CRD42017067164].

## 1. Introduction

In 2021, 38.4 million people were living with HIV worldwide and 1.5 million people became newly infected (1). The HIV pandemic remains a major global health challenge, and its extreme global genetic diversity impedes treatment and prevention efforts (2). Global temporal analysis indicates that the HIV-1 pandemic is diversifying, with increases in both the numbers of distinct HIV-1 variants and proportions of recombinant strains (3–5). Increasing diversity impacts HIV diagnosis and treatment, drug resistance, viral load measurement, transmission, disease progression, immune responses, and vaccine development (2, 6–10).

After zoonotic transmission from chimpanzees to humans in Central Africa around 1900, the HIV-1 group M epidemic rapidly diversified into distinct subtypes, designated by the letters A–D, F–H, and J–L (11, 12). HIV-1 subtypes spread across the globe throughout the 20th century, resulting in HIV-1 subtype distributions that greatly vary by region (3, 13). The genetic complexity of the HIV pandemic continues to increase over time, largely driven by the high mutation and recombination rates of the error-prone reverse transcriptase enzyme (14). Recombination occurs when an individual is co-infected with multiple strains which combine into a new variant (15). The resulting variants are designated as circulating recombinant forms (CRFs) or unique recombinant forms (URFs). CRFs, which are characterized by community spread, must be fully sequenced and found in at least three epidemiologically unlinked individuals. More than 120 distinct CRFs have been described to date, and more CRFs continue to be identified (16). URFs are unique recombinant sequences without evidence of onward transmission. The proportion of recombinants has been increasing over time, both globally and in most regions, and recombinants now constitute nearly a quarter of all HIV-1 infections globally (4). In addition to increasing the genetic complexity of the HIV pandemic, recombination may confer an evolutionary advantage, leading to altered transmission and/or virulence (17, 18).

In 2021, 70% of new HIV infections occurred within key populations and their sexual partners, though these populations account for <5% of the global population (1). It is estimated that men who have sex with men (MSM) have 28 times the risk of HIV infection relative to heterosexual (HET) adult men, female commercial sex workers (CSW) have 30 times the risk relative to other adult women, and people who inject drugs (PWID) have 35 times the risk compared to those who do not inject drugs (1). Additionally, people in areas without comprehensive blood screening are particularly vulnerable to HIV infection through transfusions with infected blood (BLOOD) (19), and children born to mothers with HIV can become infected via vertical transmission (VERT) during pregnancy, labor, delivery, or breastfeeding (20). Prior work indicates that HIV can follow a chain of transmission among these groups, spreading from PWID to CSW who transmit the virus to their HET clients. The virus can then be transmitted to the client's female sexual partner before VERT transmission of HIV infection to children (20, 21). Transmission among MSM and during blood transfusions has also played a major historical role in the spread of HIV, particularly across Asia, Europe, and North America (19, 21, 22).

Though these key populations are known to play a role in HIV transmission, it is unclear what role they play in the spread of HIV-1 recombinants. Since these populations often face difficulties accessing HIV services and have an increased risk of infection (1), potentially by multiple strains, they may be more likely to develop novel HIV strains. These recombinant strains may cross from key populations into the general population, making the overall HIV epidemic more complex.

The global proportion of HIV infections with recombinants is increasing and key populations globally account for most new HIV infections. However, there is an evidence gap regarding the global association of key populations with HIV-1 recombinants. To address this gap, we conducted a global analysis of the association between multiple key populations and HIV-1 recombinants using the largest global HIV-1 molecular epidemiology database assembled to date.

## 2. Materials and methods

### 2.1. Data collection

Data on the global distribution of HIV-1 subtypes and recombinants among key populations were obtained through a systematic literature review (PROSPERO: CRD42017067164), review of specialist journals and reports, and global survey of experts (3). We searched PubMed (29,825 citations retrieved), Embase (Ovid) (25,914 citations), CINAHL (Ebscohost) (451 citations), and Global Health (Ovid) (9,707 citations) for studies reporting HIV-1 subtyping data published from Jan 1, 1990 to Dec 31, 2015. This time period covers the period for which reliable estimates of national HIV prevalence were available. Search terms were Medical Subject Headings (MeSH) and Emtree terms, free text words, and synonyms, including “HIV,” “Subtype,” “recombinant,” “CRF,” and “URF” (Appendix pp2–5). No language or methodology filters were used. All references retrieved were combined in Endnote reference manager, and duplicates removed (Endnote X9; Clarivate Analytics, Philadelphia, PA). Authors RE, JY, LD-T and JH screened titles and abstracts, retrieved relevant full text articles, and assessed articles against the eligibility criteria. Additional published data were derived from the WHO HIV Drug Resistance Report 2012 (23), published reviews and reports on HIV diversity, and papers indexed on Scopus that referenced previous publications on global HIV-1 diversity (Appendix pp6–8). Additionally, four specialist journals (*AIDS, Journal of AIDS, Journal of Virology, AIDS Research and Human Retroviruses*) were screened for relevant articles published between January 1990 and February 2016. Using a data collection template, unpublished original HIV-1 subtyping data was collected through a global survey of members in the WHO-UNAIDS Network for HIV Isolation and Characterisation.

### 2.2. Eligibility criteria and data extraction

Studies were eligible for inclusion if they were prevalence studies of key populations living with HIV with original HIV-1 subtyping data, known country and year of sample collection (1990–2015), and a minimum of 20 participants. Studies that only contained incident infections or untyped samples were excluded. Full-length genomes or any genome segment could be used for subtyping, no minimum sequence length was specified, all online subtyping tools were accepted, and subtyping data from each included dataset was assumed to be correct.

Authors RE, JY, LD-T, and JH extracted the following information for each data set: country, city or region, sample collection year(s), study type, key population, HIV-1 subtyping method(s), and genome segment(s) analyzed. The primary outcome was the number of each key population designated by the original authors as each HIV-1 subtype (A, B, C, D, F, G, H, J, K), CRFs, and URFs. Country designation was based on where samples were taken. One subtype/CRF/URF was assigned to each participant. Subtyping methods included sequencing, heteroduplex mobility assay, and serotyping. The vast majority of data was acquired by sequencing (100% in 2010–2015), mostly of partial genome sequences, mainly pol (94.4% in 2010–2015) (3). Contributing researchers were assumed to have obtained consent from participants, and no personal identifiable information was retrieved. Formal assessment of individual study quality was not performed. Discrepancies were resolved by the senior reviewer (JH).

### 2.3. Key populations

Based on the populations specified by each study, participants were categorized as heterosexual (HET), men who have sex with men (MSM), people who inject drugs (PWID), vertical transmissions (VERT), commercial sex workers (CSW), and transfusion-associated infections (BLOOD) by author NN and confirmed by JH. Studies involving multiple key populations were assigned to the key population comprising at least 95% of data or excluded if no single key population met the 95% threshold. Studies with unspecified or indeterminate key populations were excluded. Any discrepancies or ambiguities were resolved by JH.

### 2.4. Meta-analysis

As most studies provided data on a single key population, one-stage meta-analysis of individual-participant data of different studies was performed. For logistic regression, HIV-1 variants were categorized as “Subtype” or “Recombinant” (CRF/URF). A univariate binomial logistic regression model was constructed to analyse the global association of each key population with HIV-1 recombinants. To assess the global association of key populations with CRFs and URFs separately, logistic regression was repeated using a multinomial model.

Countries were grouped into 14 regions (Appendix p9) and data were assigned to four periods: 1990–1999, 2000–2004, 2005–2009, and 2010–2015 (Appendix p10). All participants in each dataset were assigned to periods based on the midpoint year of the reported sample collection period. Datasets of which sampling years were evenly split between two periods (e.g., 2003–2006) were excluded from time-stratified analyses. To assess temporal and geographic differences in the associations with recombinants, the binomial logistic model was separately stratified into subgroups by time period and region.

For all logistic models, “Subtype” was used as the reference group. Odds Ratios (ORs) were reported with 95% confidence intervals. In the Appendix, pairwise ORs are reported globally and for each region (pp11–13) and period subgroup analyses were repeated for the multinomial logistic regression model (pp14). Statistical analyses were performed using STATA 17.0 (StataCorp LLC, College Station, TX). This systematic review is reported according to the PRISMA guidelines, as applicable.

## 3. Results

### 3.1. Data collection

**Figure 1:**
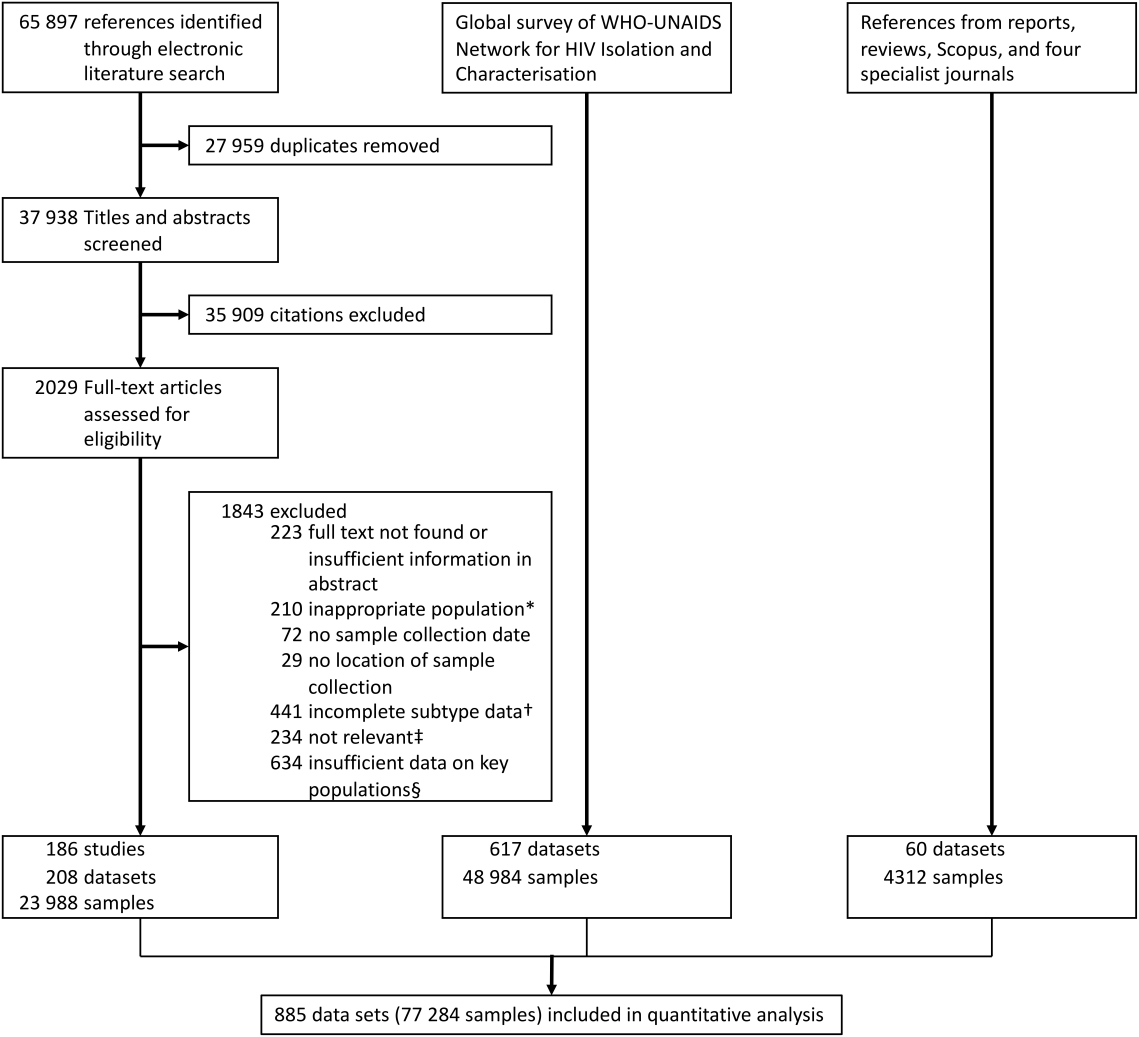
Data collection flowchart. *For example, HIV-positive immigrants only. ^†^For example, data only provided for subtype B and non-B participants. ^‡^For example, subtypes referred to disease states, not HIV subtypes. ^§^For example, HIV subtyping data could not be assigned to a specific key population, as multiple key populations were present in the study.

A total of 885 datasets including 77,284 participants from key populations from 83 countries were included (Figure 1). The systematic literature search yielded 208 datasets comprising 23,988 participants. Six hundred and seventeen datasets with 48,984 participants were collected from the global survey, and 60 datasets with 4,312 participants were obtained from other published sources.

Most included participants were heterosexual people (58.2%) and MSM (25.7%), with smaller proportions of participants representing PWID (8.1%), VERT (5.3%), CSW (2.2%), and BLOOD (0.6%) (Table 1). Data from the HET population was the largest in each time period, while there was no data for CSW in the most recent period. Most participants were derived from Western and central Europe, and North America (WCENA) followed by East, West, and Southern Africa. HET data was available in every geographic region while all BLOOD participants were derived from East Asia and WCENA. HET was selected as the reference group as it was the only population represented in all regions and time periods.

**Table 1:** Data collection on key populations and HIV-1 subtypes and recombinants, 1990–2015.

	**HET**	**MSM**	**PWID**	**VERT**	**CSW**	**BLOOD**	**Total**
**Number of datasets**							
**GLOBAL (1990–2015)**	**538 (60.8%)**	**110 (12.4%)**	**98 (11.1%)**	**86 (9.7%)**	**20 (2.3%)**	**33 (3.7%)**	**885 (100%)**
1990–1999	109 (56.5%)	18 (9.3%)	36 (18.7%)	18 (9.3%)	4 (2.1%)	8 (4.1%)	193 (100%)
2000–2004	106 (55.5%)	22 (11.5%)	28 (14.7%)	16 (8.4%)	10 (5.2%)	9 (4.7%)	191 (100%)
2005–2009	230 (73.0%)	35 (11.1%)	17 (5.4%)	19 (6.0%)	6 (1.9%)	8 (2.5%)	315 (100%)
2010–2015	86 (49.1%)	32 (18.3%)	17 (9.7%)	32 (18.3%)	0 (0.0%)	8 (4.6%)	175 (100%)
Caribbean	10 (76.9%)	3 (23.1%)	0 (0.0%)	0 (0.0%)	0 (0.0%)	0 (0.0%)	13 (100%)
Latin America	18 (40.0%)	8 (17.8%)	4 (8.9%)	12 (26.7%)	3 (6.7%)	0 (0.0%)	45 (100%)
Western and central Europe, and North America (WCENA)	56 (23.5%)	66 (27.7%)	44 (18.5%)	40 (16.8%)	0 (0.0%)	32 (13.4%)	238 (100%)
Eastern Europe and central Asia (EECA)	6 (27.3%)	2 (9.1%)	13 (59.1%)	1 (4.5%)	0 (0.0%)	0 (0.0%)	22 (100%)
South Asia	8 (61.5%)	0 (0.0%)	2 (15.4%)	0 (0.0%)	3 (23.1%)	0 (0.0%)	13 (100%)
Southeast Asia (SE Asia)	28 (50.0%)	1 (1.8%)	21 (37.5%)	5 (8.9%)	1 (1.8%)	0 (0.0%)	56 (100%)
East Asia	2 (6.2%)	16 (50.0%)	12 (37.5%)	1 (3.1%)	0 (0.0%)	1 (3.1%)	32 (100%)
Oceania	3 (30.0%)	7 (70.0%)	0 (0.0%)	0 (0.0%)	0 (0.0%)	0 (0.0%)	10 (100%)
Middle East and North Africa (MENA)	1 (50.0%)	0 (0.0%)	1 (50.0%)	0 (0.0%)	0 (0.0%)	0 (0.0%)	2 (100%)
West Africa	137 (90.1%)	4 (2.6%)	0 (0.0%)	6 (3.9%)	5 (3.3%)	0 (0.0%)	152 (100%)
East Africa	91 (83.5%)	0 (0.0%)	1 (0.9%)	9 (8.3%)	8 (7.3%)	0 (0.0%)	109 (100%)
Ethiopia	7 (100.0%)	0 (0.0%)	0 (0.0%)	0 (0.0%)	0 (0.0%)	0 (0.0%)	7 (100%)
Central Africa	39 (95.1%)	0 (0.0%)	0 (0.0%)	2 (4.9%)	0 (0.0%)	0 (0.0%)	41 (100%)
Southern Africa	132 (91.0%)	3 (2.1%)	0 (0.0%)	10 (6.9%)	0 (0.0%)	0 (0.0%)	145 (100%)
**Number of participants**							
**GLOBAL (1990–2015)**	**44,952 (58.2%)**	**19,835 (25.7%)**	**6,236 (8.1%)**	**4,128 (5.3%)**	**1,685 (2.2%)**	**448 (0.6%)**	**77,284 (100%)**
1990–1999	10,021 (71.1%)	1,071 (7.6%)	2,333 (16.5%)	370 (2.6%)	277 (2.0%)	27 (0.2%)	14,099 (100%)
2000–2004	9,369 (57.5%)	3,602 (22.1%)	1,829 (11.2%)	406 (2.5%)	965 (5.9%)	131 (0.8%)	16,302 (100%)
2005–2009	13,845 (62.1%)	5,487 (24.6%)	1,510 (6.8%)	754 (3.4%)	443 (2.0%)	242 (1.1%)	22,281 (100%)
2010–2015	10,768 (46.4%)	9,293 (40.0%)	564 (2.4%)	2,539 (10.9%)	0 (0.0%)	48 (0.2%)	23,212 (100%)
Caribbean	662 (61.1%)	421 (38.9%)	0 (0.0%)	0 (0.0%)	0 (0.0%)	0 (0.0%)	1,083 (100%)
Latin America	3,146 (60.3%)	883 (16.9%)	241 (4.6%)	828 (15.9%)	121 (2.3%)	0 (0.0%)	5,219 (100%)
Western and central Europe, and North America (WCENA)	6,485 (31.8%)	11,726 (57.5%)	1,390 (6.8%)	516 (2.5%)	0 (0.0%)	269 (1.3%)	20,386 (100%)
Eastern Europe and central Asia (EECA)	263 (25.3%)	75 (7.2%)	694 (66.7%)	8 (0.8%)	0 (0.0%)	0 (0.0%)	1,040 (100%)
South Asia	541 (73.6%)	0 (0.0%)	80 (10.9%)	0 (0.0%)	114 (15.5%)	0 (0.0%)	735 (100%)
Southeast Asia (SE Asia)	3,649 (54.1%)	425 (6.3%)	2,195 (32.5%)	319 (4.7%)	157 (2.3%)	0 (0.0%)	6,745 (100%)
East Asia	763 (10.3%)	4,885 (66.1%)	1,537 (20.8%)	22 (0.3%)	0 (0.0%)	179 (2.4%)	7,386 (100%)
Oceania	93 (10.1%)	826 (89.9%)	0 (0.0%)	0 (0.0%)	0 (0.0%)	0 (0.0%)	919 (100%)
Middle East and North Africa (MENA)	71 (64.0%)	0 (0.0%)	40 (36.0%)	0 (0.0%)	0 (0.0%)	0 (0.0%)	111 (100%)
West Africa	9,286 (91.1%)	333 (3.3%)	0 (0.0%)	110 (1.1%)	465 (4.6%)	0 (0.0%)	10,194 (100%)
East Africa	9,347 (85.3%)	0 (0.0%)	59 (0.5%)	730 (6.7%)	828 (7.6%)	0 (0.0%)	10,964 (100%)
Ethiopia	230 (100.0%)	0 (0.0%)	0 (0.0%)	0 (0.0%)	0 (0.0%)	0 (0.0%)	230 (100%)
Central Africa	2,673 (93.5%)	0 (0.0%)	0 (0.0%)	186 (6.5%)	0 (0.0%)	0 (0.0%)	2,859 (100%)
Southern Africa	7,743 (82.3%)	261 (2.8%)	0 (0.0%)	1,409 (15.0%)	0 (0.0%)	0 (0.0%)	9,413 (100%)

BLOOD, blood/plasma transfusion associated infections; CSW, commercial sex workers; HET, heterosexual; MSM, men who have sex with men; PWID, people who inject drugs; VERT, vertical transmission (mother to child).

### 3.2. Global association of key populations with recombinants

The global distribution of HIV-1 subtypes, CRFs and URFs among key populations in 1990–2015 is shown in Table 2. During 1990–2015, the largest proportion of recombinant infections was found among PWID (52.8%) and CSW (40.5%), followed by HET (30.1%), MSM (29.4%), VERT (19.9%), and BLOOD (15.6%). PWID had the highest proportion of CRFs (49.1%) and CSW had the highest proportion of URFs (17.6%). The proportion of recombinant infections grew consistently across periods for HET, MSM, and BLOOD and across the first three periods for PWID. Global proportions of individual CRFs for each key population are included in the Appendix p16.

**Table 2:** Global distribution of HIV-1 subtypes, CRFs, and URFs among key populations, 1990–2015.

	**HIV-1 subtypes**									**CRFs**			**URFs**	Total CRFs^*^	**Total recombinants^†^**	**Total^‡^**
	**A**	**B**	**C**	**D**	**F**	**G**	**H**	**J**	**K**	**CRF01_AE**	**CRF02_AG**	**Other**				
**Global (1990–2015)**																
HET	8,340 (18.6%)	5,889 (13.1%)	11,408 (25.4%)	3,222 (7.2%)	580 (1.3%)	1,729 (3.8%)	182 (0.4%)	64 (0.1%)	22 (0.0%)	4,140 (9.2%)	4,995 (11.1%)	1,807 (4%)	2,574 (5.7%)	10,942 (24.3%)	13,516 (30.1%)	44,952 (100%)
MSM	157 (0.8%)	12,937 (65.2%)	561 (2.8%)	12 (0.1%)	219 (1.1%)	104 (0.5%)	4 (0.0%)	1 (0.0%)	7 (0.0%)	3,154 (15.9%)	446 (2.2%)	1,730 (8.7%)	503 (2.5%)	5,330 (26.9%)	5,833 (29.4%)	19,835 (100%)
PWID	794 (12.7%)	1,846 (29.6%)	169 (2.7%)	8 (0.1%)	116 (1.9%)	13 (0.2%)	0 (0.0%)	0 (0.0%)	0 (0.0%)	1,363 (21.9%)	8 (0.1%)	1,691 (27.1%)	228 (3.7%)	3,062 (49.1%)	3,290 (52.8%)	6,236 (100%)
VERT	578 (14%)	824 (20%)	1,627 (39.4%)	152 (3.7%)	71 (1.7%)	45 (1.1%)	5 (0.1%)	3 (0.1%)	0 (0.0%)	328 (7.9%)	136 (3.3%)	96 (2.3%)	263 (6.4%)	560 (13.6%)	823 (19.9%)	4,128 (100%)
CSW	439 (26.1%)	47 (2.8%)	367 (21.8%)	72 (4.3%)	19 (1.1%)	56 (3.3%)	2 (0.1%)	0 (0.0%)	0 (0.0%)	155 (9.2%)	196 (11.6%)	36 (2.1%)	296 (17.6%)	387 (23%)	683 (40.5%)	1,685 (100%)
BLOOD	11 (2.5%)	270 (60.3%)	19 (4.2%)	5 (1.1%)	70 (15.6%)	3 (0.7%)	0 (0.0%)	0 (0.0%)	0 (0.0%)	18 (4%)	25 (5.6%)	13 (2.9%)	14 (3.1%)	56 (12.5%)	70 (15.6%)	448 (100%)
**1990–1999**												
HET	4,229 (42.2%)	462 (4.6%)	1,832 (18.3%)	1,607 (16.0%)	239 (2.4%)	321 (3.2%)	80 (0.8%)	24 (0.2%)	8 (0.1%)	609 (6.1%)	245 (2.4%)	38 (0.4%)	327 (3.3%)	892 (8.9%)	1,219 (12.2%)	10,021 (100%)
MSM	3 (0.3%)	1,041 (97.2%)	17 (1.6%)	1 (0.1%)	3 (0.3%)	0 (0.0%)	0 (0.0%)	0 (0.0%)	0 (0.0%)	3 (0.3%)	0 (0.0%)	0 (0.0%)	3 (0.3%)	3 (0.3%)	6 (0.6%)	1,071 (100%)
PWID	383 (16.4%)	979 (42.0%)	30 (1.3%)	0 (0.0%)	0 (0.0%)	0 (0.0%)	0 (0.0%)	0 (0.0%)	0 (0.0%)	792 (33.9%)	0 (0.0%)	138 (5.9%)	11 (0.5%)	930 (39.9%)	941 (40.3%)	2,333 (100%)
VERT	68 (18.4%)	86 (23.2%)	73 (19.7%)	32 (8.6%)	11 (3.0%)	4 (1.1%)	0 (0.0%)	0 (0.0%)	0 (0.0%)	43 (11.6%)	3 (0.8%)	16 (4.3%)	34 (9.2%)	62 (16.8%)	96 (25.9%)	370 (100%)
CSW	72 (26.0%)	3 (1.1%)	35 (12.6%)	1 (0.4%)	0 (0.0%)	12 (4.3%)	0 (0.0%)	0 (0.0%)	0 (0.0%)	154 (55.6%)	0 (0.0%)	0 (0.0%)	0 (0.0%)	154 (55.6%)	154 (55.6%)	277 (100%)
BLOOD	1 (3.7%)	25 (92.6%)	1 (3.7%)	0 (0.0%)	0 (0.0%)	0 (0.0%)	0 (0.0%)	0 (0.0%)	0 (0.0%)	0 (0.0%)	0 (0.0%)	0 (0.0%)	0 (0.0%)	0 (0.0%)	0 (0.0%)	27 (100%)
**2000–2004**												
HET	1,351 (14.4%)	1,143 (12.2%)	2,840 (30.3%)	714 (7.6%)	82 (0.9%)	232 (2.5%)	11 (0.1%)	10 (0.1%)	3 (0.0%)	1,164 (12.4%)	1,024 (10.9%)	335 (3.6%)	460 (4.9%)	2,523 (26.9%)	2,983 (31.8%)	9,369 (100%)
MSM	22 (0.6%)	3,399 (94.4%)	82 (2.3%)	0 (0.0%)	12 (0.3%)	10 (0.3%)	0 (0.0%)	0 (0.0%)	0 (0.0%)	28 (0.8%)	25 (0.7%)	3 (0.1%)	21 (0.6%)	56 (1.6%)	77 (2.1%)	3,602 (100%)
PWID	246 (13.4%)	568 (31.1%)	21 (1.1%)	0 (0.0%)	17 (0.9%)	6 (0.3%)	0 (0.0%)	0 (0.0%)	0 (0.0%)	340 (18.6%)	5 (0.3%)	488 (26.7%)	138 (7.5%)	833 (45.5%)	971 (53.1%)	1,829 (100%)
VERT	85 (20.9%)	215 (53.0%)	31 (7.6%)	11 (2.7%)	16 (3.9%)	1 (0.2%)	0 (0.0%)	0 (0.0%)	0 (0.0%)	0 (0.0%)	25 (6.2%)	3 (0.7%)	19 (4.7%)	28 (6.9%)	47 (11.6%)	406 (100%)
CSW	126 (13.1%)	21 (2.2%)	291 (30.2%)	25 (2.6%)	18 (1.9%)	38 (3.9%)	0 (0.0%)	2 (0.2%)	0 (0.0%)	1 (0.1%)	189 (19.6%)	35 (3.6%)	219 (22.7%)	225 (23.3%)	444 (46%)	965 (100%)
BLOOD	5 (3.8%)	35 (26.7%)	7 (5.3%)	1 (0.8%)	70 (53.4%)	1 (0.8%)	0 (0.0%)	0 (0.0%)	0 (0.0%)	5 (3.8%)	5 (3.8%)	0 (0.0%)	2 (1.5%)	10 (7.6%)	12 (9.2%)	131 (100%)
**2005–2009**												
HET	2,030 (14.7%)	1,769 (12.8%)	4,194 (30.3%)	691 (5.0%)	154 (1.1%)	473 (3.4%)	78 (0.6%)	26 (0.2%)	5 (0.0%)	842 (6.1%)	1,757 (12.7%)	848 (6.1%)	978 (7.1%)	3,447 (24.9%)	4,425 (32%)	13,845 (100%)
MSM	37 (0.7%)	4,218 (76.9%)	105 (1.9%)	2 (0.0%)	20 (0.4%)	13 (0.2%)	1 (0.0%)	1 (0.0%)	0 (0.0%)	740 (13.5%)	87 (1.6%)	134 (2.4%)	129 (2.4%)	961 (17.5%)	1,090 (19.9%)	5,487 (100%)
PWID	79 (5.2%)	209 (13.8%)	91 (6.0%)	3 (0.2%)	5 (0.3%)	2 (0.1%)	0 (0.0%)	0 (0.0%)	0 (0.0%)	219 (14.5%)	1 (0.1%)	849 (56.2%)	52 (3.4%)	1,069 (70.8%)	1,121 (74.2%)	1,510 (100%)
VERT	35 (4.6%)	260 (34.5%)	181 (24.0%)	10 (1.3%)	1 (0.1%)	5 (0.7%)	1 (0.1%)	0 (0.0%)	0 (0.0%)	146 (19.4%)	19 (2.5%)	14 (1.9%)	82 (10.9%)	179 (23.7%)	261 (34.6%)	754 (100%)
CSW	241 (54.4%)	23 (5.2%)	41 (9.3%)	46 (10.4%)	1 (0.2%)	6 (1.4%)	0 (0.0%)	0 (0.0%)	0 (0.0%)	0 (0.0%)	7 (1.6%)	1 (0.2%)	77 (17.4%)	8 (1.8%)	85 (19.2%)	443 (100%)
BLOOD	1 (0.4%)	189 (78.1%)	8 (3.3%)	4 (1.7%)	0 (0.0%)	0 (0.0%)	0 (0.0%)	0 (0.0%)	0 (0.0%)	12 (5.0%)	10 (4.1%)	10 (4.1%)	8 (3.3%)	32 (13.2%)	40 (16.5%)	242 (100%)
**2010–2015**												
HET	553 (5.1%)	2,505 (23.3%)	2,526 (23.5%)	164 (1.5%)	100 (0.9%)	548 (5.1%)	10 (0.1%)	2 (0.0%)	5 (0.0%)	1,344 (12.5%)	1,789 (16.6%)	553 (5.1%)	669 (6.2%)	3,686 (34.2%)	4,355 (40.4%)	10,768 (100%)
MSM	91 (1.0%)	4,200 (45.2%)	159 (1.7%)	9 (0.1%)	180 (1.9%)	73 (0.8%)	1 (0.0%)	0 (0.0%)	7 (0.1%)	2,383 (25.6%)	334 (3.6%)	1,517 (16.3%)	339 (3.6%)	4,234 (45.6%)	4,573 (49.2%)	9,293 (100%)
PWID	86 (15.2%)	90 (16.0%)	27 (4.8%)	5 (0.9%)	94 (16.7%)	5 (0.9%)	0 (0.0%)	0 (0.0%)	0 (0.0%)	12 (2.1%)	2 (0.4%)	216 (38.3%)	27 (4.8%)	230 (40.8%)	257 (45.6%)	564 (100%)
VERT	390 (15.4%)	252 (9.9%)	1,303 (51.3%)	99 (3.9%)	42 (1.7%)	35 (1.4%)	4 (0.2%)	3 (0.1%)	0 (0.0%)	139 (5.5%)	89 (3.5%)	63 (2.5%)	120 (4.7%)	291 (11.5%)	411 (16.2%)	2,539 (100%)
BLOOD	4 (8.3%)	21 (43.8%)	3 (6.2%)	0 (0.0%)	0 (0.0%)	2 (4.2%)	0 (0.0%)	0 (0.0%)	0 (0.0%)	1 (2.1%)	10 (20.8%)	3 (6.2%)	4 (8.3%)	14 (29.2%)	18 (37.5%)	48 (100%)

Global distribution of HIV-1 subtypes, CRFs, and URFs within key populations in 1990–2015 and each of four time periods (1990–1999, 2000–2004, 2005–2009, 2010–2015).

^*^Total CRFs is the sum of CRF01_AE, CRF02_AG, and Other CRFs.

^†^Total recombinants is the sum of total CRFs and URFs.

^‡^Total is the sum of total recombinants and all HIV-1 subtypes.

BLOOD, blood/plasma transfusion associated infections; CRF, circulating recombinant form; CSW, commercial sex workers; HET, heterosexual; MSM, men who have sex with men; PWID, people who inject drugs; URF, unique recombinant form; VERT, vertical transmission (mother to child).

**Figure 2:**
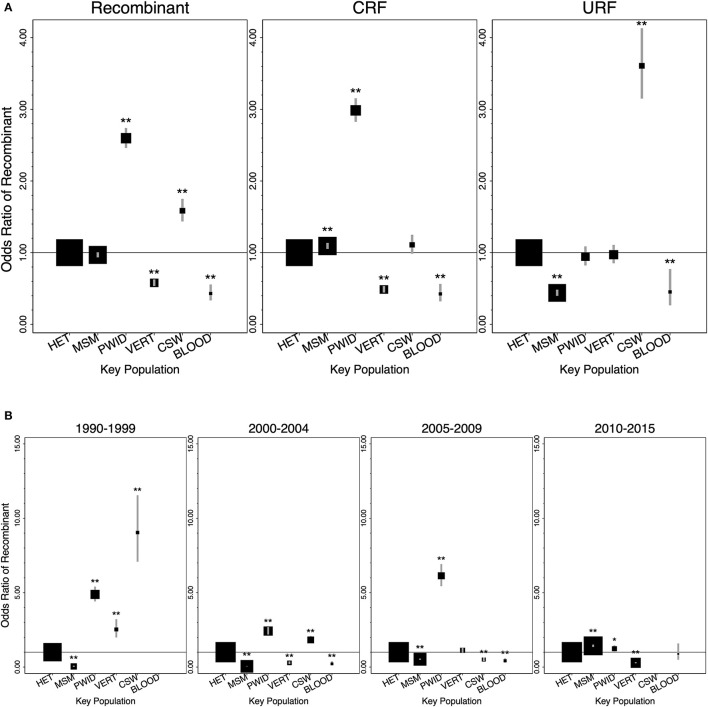
Global associations of key populations with HIV-1 recombinants, CRFs, and URFs relative to heterosexual people (1990–2015). **(A)** Odds ratios for HIV-1 recombinants, CRFs, and URFs, compared to HIV-1 subtypes, of key populations relative to heterosexual people (1990–2015). **(B)** Odds ratios for HIV-1 recombinants, compared to HIV-1 subtypes, of key populations relative to heterosexual people in each time period (1990–1999, 2000–2004, 2005–2009, 2010–2015). No data on recombinants was available for BLOOD in 1990–1999, and no data was available for CSW in 2010–2015. Error bars represent the 95% confidence intervals. Square areas are proportional to the number of participants in each key population analyzed. Odds ratios and 95% CI are provided in the Appendix p15 (*P < 0.05, **P < 0.01). BLOOD, blood/plasma transfusion associated infections; CRF, circulating recombinant form; CSW, commercial sex workers; HET, Heterosexual; MSM, men who have sex with men; PWID, people who inject drugs; URF, Unique Recombinant Form; VERT, vertical transmission (mother to child).

PWID were associated with the greatest odds of recombinants relative to all other key populations (Figure 2A; Table 3). Relative to HET, PWID and CSW were associated with increased odds of recombinants [OR 2.6 (95% CI 2.46–2.74) and 1.59 (1.44–1.75)], while VERT and BLOOD were associated with decreased odds [0.58 (0.54–0.63) and 0.43 (0.33–0.56)]. MSM did not have a significant association with recombinants relative to HET [0.97 (0.93–1.01)] (Figure 2A; Table 3).

**Table 3:** Regional associations of HIV-1 recombinants, CRFs, and URFs with key populations relative to heterosexual people, 1990–2015.

	GLOBAL^*^	**Latin America**	**Western and central Europe, and North America**	**Eastern Europe and central Asia**	**South Asia**	**Southeast Asia**	**East Asia**	**West Africa**	**East Africa**	**Central Africa**	**Southern Africa**
**Recombinants**											
MSM	0.97 (0.93–1.01)	**0.46 (0.31–0.67)**	**0.18 (0.16–0.20)**	–	–	0.67 (0.42–1.05)	**1.94 (1.60–2.34)**	**1.44 (1.14–1.82)**	–	–	1.13 (0.35–3.60)
PWID	**2.60 (2.46–2.74)**	**4.16 (3.02–5.74)**	0.94 (0.82–1.08)	**19.98 (6.30–63.34)**	4.18 (0.98–17.83)	**0.06 (0.05–0.07)**	**3.20 (2.50–4.09)**	–	**–**	–	–
VERT	**0.58 (0.54–0.63)**	**3.10 (2.50–3.85)**	**0.69 (0.55–0.88)**	–	–	**12.12 (1.69–86.98)**	0.79 (0.30–2.04)	**3.39 (2.04–5.63)**	1.08 (0.85–1.38)	0.80 (0.58–1.10)	**0.14 (0.03–0.56)**
CSW	**1.59 (1.44–1.75)**	**15.58 (10.63–22.85)**	–	–	–	1.96 (0.62–6.21)	–	**0.68 (0.56–0.82)**	**3.36 (2.85–3.97)**	–	–
BLOOD	**0.43 (0.33–0.56)**	–	**0.70 (0.51–0.96)**	–	–	–	**0.04 (0.02–0.06)**	–	–	–	–
**CRFs**											
MSM	**1.09 (1.05–1.14)**	–	**0.18 (0.16–0.20)**	–	–	**0.59 (0.37–0.92)**	**1.92 (1.59–2.32)**	1.21 (0.94–1.55)	–	–	–
PWID	**2.99 (2.83–3.16)**	**2.72 (1.45–5.11)**	1.03 (0.89–1.19)	**54.89 (7.62–395.26)**	–	**0.05 (0.05–0.07)**	**3.26 (2.55–4.18)**	–	–	–	–
VERT	**0.49 (0.44–0.53)**	**1.85 (1.20–2.86)**	**0.74 (0.58–0.94)**	–	–	**12.28 (1.71–88.12)**	0.77 (0.29–2.02)	**3.40 (2.03–5.68)**	0.58 (0.24–1.43)	0.72 (0.47–1.09)	0.29 (0.07–1.22)
CSW	1.11 (0.98–1.25)	–	–	–	–	1.98 (0.62–6.29)	–	**0.77 (0.64–0.93)**	–	–	–
BLOOD	**0.43 (0.32–0.56)**	–	**0.65 (0.45–0.92)**	–	–	–	**0.04 (0.02–0.06)**	–	–	–	–
**URFs**											
MSM	**0.44 (0.40–0.48)**	**0.66 (0.45–0.97)**	**0.18 (0.15–0.23)**	–	–	**11.98 (7.91–18.13)**	1.19 (0.78–1.82)	**2.44 (1.80–3.30)**	–	–	2.12 (0.65–6.89)
PWID	0.95 (0.82–1.09)	**4.80 (3.36–6.85)**	**0.52 (0.35–0.75)**	2.54 (0.56–11.52)	**5.22 (1.15–23.78)**	**6.61 (4.62–9.47)**	**0.53 (0.31–0.89)**	–	–	–	–
VERT	0.97 (0.85–1.11)	**3.65 (2.86–4.66)**	**0.51 (0.28–0.91)**	–	–	–	1.50 (0.19–11.84)	0.98 (0.53–1.80)	1.15 (0.89–1.47)	0.90 (0.59–1.35)	–
CSW	**3.61 (3.15–4.13)**	**22.46 (15.17–33.23)**	–	–	–	–	–	**0.06 (0.01–0.23)**	**3.81 (3.22–4.51)**	–	–
BLOOD	**0.45 (0.26–0.77)**	–	0.97 (0.54–1.76)	–	–	–	2.37 (0.52–10.76)	–	–	–	–

Odds ratios and 95% confidence intervals by region for binomial/multinomial logistic regression between key populations and recombinants, CRFs, and URFs compared to HIV-1 subtypes. Significant results are in bold.

^*^Regions with insufficient data on key populations to fit a logistic regression model for recombinants (Caribbean, Ethiopia, Middle East and North Africa, Oceania) were included in the global association, but not as independent regions.

Additional data on global and regional odds ratios of key populations and HIV-1 recombinants is included in the Appendix pp11–13.

BLOOD, blood/plasma transfusion associated infections; CRF, circulating recombinant form; CSW, commercial sex workers; HET, heterosexual; MSM, men who have sex with men; PWID, people who inject drugs; URF, unique recombinant form; VERT, vertical transmission (mother to child).

Relative to HET, independent associations of each key population with CRFs and URFs varied substantially (Figure 2A; Appendix p15). PWID were significantly associated with increased odds of CRFs [2.99 (2.83–3.16)], while CSW were significantly associated with increased odds of URFs [3.61 (3.15–4.13)]. VERT was associated with decreased odds of CRFs [0.49 (0.44–0.53)], and BLOOD was associated with decreased odds of both CRFs and URFs [0.43 (0.32–0.56) and 0.45 (0.26–0.77)]. MSM were associated with slightly increased odds of CRFs [1.09 (1.05–1.14)], but decreased odds of URFs [0.44 (0.40–0.48)] (Figure 2A; Appendix p15).

Relative to HET, the strength of associations with recombinants across time differed by key population (Figure 2B; Appendix pp14, 15). PWID were associated with increased odds of recombinants across all periods. CSW were initially associated with increased odds of recombinants, but the strength of the association decreased with time before leading to decreased odds in the 2005–2009 period. VERT alternated from increased to decreased odds of recombinants across time. BLOOD was associated with decreased odds of recombinants during 2000–2009 but was not significant in the most recent period. MSM had decreased odds of recombinants during 1990–2009 but trended upwards and were associated with increased odds in 2010–2015 [1.43 (1.35–1.51)].

### 3.3. Regional association of key populations with recombinants

The regional distribution of HIV-1 subtypes, CRFs, and URFs for each key population is included in the Appendix pp17–19. The association of key populations with recombinants varied by region (Table 3). Compared to HET, PWID had the greatest odds of recombinants in Eastern Europe and central Asia [EECA; OR 19.98 (95% CI 6.30–63.34)], followed by Latin America [4.16 (3.02–5.74)] and East Asia [3.20 (2.50–4.09)]. PWID were significantly associated with CRFs in EECA [54.89 (7.62–395.26)] and East Asia [3.26 (2.55–4.18)], and both CRFs and URFs in Latin America [2.72 (1.45–5.11) and 4.80 (3.36–6.85)]. In contrast, PWID had a strong negative association with recombinants in Southeast Asia [0.06 (0.05–0.07)].

CSW had the largest odds of recombinants in Latin America [15.58 (10.63–22.85)] and East Africa [3.36 (2.85–3.97)]. In both regions, CSW were significantly associated with URFs [22.46 (15.17–33.23) and 3.81 (3.22–4.51)]. However, in West Africa, CSW had decreased odds of recombinants [0.68 (0.56–0.82)], CRFs [0.77 (0.64–0.93)], and URFs [0.06 (0.01–0.23)].

VERT was associated with decreased odds of recombinants in Southern Africa [0.14 (0.03–0.56)] and WCENA [0.69 (0.55–0.88)]. VERT was not independently associated with CRFs or URFs in Southern Africa, but had decreased odds of both CRFs [0.74 (0.58–0.94)] and URFs [0.51 (0.28–0.91)] in WCENA. In Latin America [3.10 (2.50–3.85)], West Africa [3.39 (2.04–5.63)], and SE Asia [12.12 (1.69–86.98)] VERT was associated with increased odds of recombinants. In Latin America, VERT was significantly associated with increased odds of both CRFs [1.85 (1.20–2.86)] and URFs [3.65 (2.86–4.66)], but it was only associated with increased odds of CRFs in West Africa [3.40 (2.03–5.68)] and SE Asia [12.28 (1.71–88.12)].

BLOOD was associated with decreased odds of recombinants in East Asia [0.04 (0.02–0.06)] and WCENA [0.70 (0.51–0.96)]. In both regions, BLOOD was also associated with decreased odds of CRFs [0.04 (0.02–0.06) and 0.65 (0.45–0.92)].

In East Asia, MSM were associated with increased odds of recombinants [1.94 (1.60–2.34)] and CRFs [1.92 (1.59–2,32)]. In West Africa, MSM were associated with increased odds of recombinants [1.44 (1.14–1.82)] and URFs [2.44 (1.80–3.30)]. In Latin America and WCENA, MSM had decreased odds of recombinants [0.46 (0.31–0.67) and 0.18 (0.16–0.20)] and URFs [0.66 (0.45–0.97) and 0.18 (0.15–0.23)].

## 4. Discussion

A strong association between PWID and recombinants and CRFs was observed globally across all periods and in most regions. Only in SE Asia, where CRF01_AE has a prevalence of ~70–80% (3), were PWID associated with decreased odds of recombinant strains. The prevalence of recombinant epidemics among PWID in most regions, where “pure” HIV-1 subtypes are typically the most prevalent overall (3), and subtype-based epidemics among PWID in SE Asia, where recombinant strains are highly prevalent, indicates that HIV-1 circulates among PWID via transmission networks distinct from the HET population. This finding extends previous studies suggesting that HIV is transmitted among independent PWID networks across multiple continents (24, 25). Furthermore, the association with recombinants across all periods indicates that PWID play a major role in the global diversification of HIV-1.

CSW were associated with increased odds of recombinants and URFs, particularly in the periods 1990–1999 and 2000–2004. Across East Africa and Latin America, CSW were significantly more likely to be infected with URFs than the HET population. These findings highlight that novel HIV strains frequently arise within the CSW population. URFs arise independently and lack evidence of transmission, minimizing the likelihood that the observed association is due to reverse causation. The decreased odds of CRFs in West Africa may be related to the high prevalence of CRF02_AG (3), similarly to the case of PWID and CRF01_AE in SE Asia. Additional data is required to identify factors contributing to the diminishing global association of CSWs with recombinants across time.

Though VERT was associated with decreased odds of recombinants and CRFs, results greatly varied across times and regions. While biological differences in recombinant strains may cause increased rates of vertical transmission relative to “pure” subtypes (26), high levels of heterogeneity indicate that VERT is not a major driver of increasing HIV-1 diversity.

BLOOD was associated with decreased odds of recombinants and CRFs in both East Asia and WCENA. Particularly in East Asia, where CRF01_AE is highly prevalent, blood transfusion recipients were significantly less likely to have a recombinant strain of HIV than the heterosexual population, which may reflect the geographical origins of the blood donor base. However, the small number of datasets means that the observed association is subject to limitations of power and representativeness. Additional data is required to clarify the association between BLOOD and recombinants.

MSM did not have a significant global association with recombinants overall, likely due to a positive association with CRFs and negative association with URFs. The positive association between MSM and CRFs was strongest in East Asia where the prevalence of CRFs has grown from 25.9 to 75.5% during 1990–2015 (3). Within this region, MSM had nearly double the odds of CRFs as HET, indicating that MSM may be at the forefront of the growing epidemic across East Asia. Similar results were seen in West Africa where the proportion of URFs grew from 3.4 to 15.5% over the same period (3), and findings indicated that MSM had 2.44 times the odds of being infected with URFs. These associations suggest that MSM likely play a major role in the spread of new strains in some regions. Despite an overall association with recombinants that was not significant and historical associations with HIV-1 subtype B (27), the significant association in 2010–2015 and increasing trend across time indicate that MSM may be associated with an increased risk of recombinants.

A key strength of this study is its unprecedented large size, including 77,284 participants from 83 countries, collected from key populations globally during 1990–2015. To our knowledge, this is the first comprehensive analysis of the association between key populations and HIV-1 subtypes and recombinants at a global and regional level. Additionally, data was collected through both a literature search and a global survey, with the inclusion of unpublished data enabling increased regional coverage and improved coverage of recent time periods.

The study also had some limitations. Estimates of associations of key populations with HIV-1 variants are dependent on the underlying data. There was notable variation in coverage by key population, geographic region, and time period. Although the numbers of participants were generally high, the limited number of datasets included from BLOOD means that results must be interpreted with caution. Conclusions could not be independently drawn for any key populations in the Caribbean, Ethiopia, Oceania, and Middle East and North Africa (MENA) due to persistent data gaps that have been previously noted (1, 28). Similarly, the absence of data for certain key populations in some regions (e.g., MSM data in Central Africa, East Africa, and MENA) may reflect limited access to healthcare due to sociolegal restrictions (29). Most data were not drawn from nationally representative surveys and we were unable to weigh country-level data according to relative numbers of people of key populations living with HIV in each country, as comprehensive global data on key populations is not available. Hence, reported distributions of HIV-1 variants should not be interpreted as representative of key populations in each region or globally. As HIV subtyping data for most studies was primarily based on *pol* sequencing rather than the whole genome (3), recombination outside of this genome region was likely missed, leading to an underestimation of recombinants. Seventy four CRFs were described at the time of data collection (up until 2015), contributing to the discrepancy between the 48 CRFs identified within the datasets contributing to this study and the >120 CRFs that have been described to date (16). Findings could be subject to bias due to heterogeneity in study design, inclusion/exclusion criteria, subtyping methods, and rates of treatment and migration across regions. In particular, differences in participant recruitment and the definition of key populations between studies could affect observed associations with recombinants. Lastly, insufficient data was available to conduct analysis for transgender women.

Among key populations, increased risk of HIV infection, potentially by multiple strains, and difficulty accessing treatment, potentially leading to increased viral loads, may contribute to the formation and onward spread of HIV-1 recombinants (18, 30). The increasing diversity of the HIV pandemic has implications across diagnosis, treatment, and prevention (2, 6–10). Efforts to prevent the spread of novel HIV strains should consider approaches for key populations such as PWID, CSW, and MSM that are at increased risk of developing and transmitting recombinants. In the case of PWID, this may require prevention-based approaches such as distribution of sterile injection equipment (31, 32), opiate substitution treatment (33), and increased access to antiretroviral therapies (34). For CSW and MSM, prevention efforts should focus on increasing availability of the dapivirine vaginal ring for cisgender women, oral TDF-based pre-exposure prophylaxis (PrEP), and long-acting injectable cabotegravir (1, 35, 36). Increased HIV testing among key populations will help detect and treat new HIV infections early. These efforts can help limit the spread of traits from newly-emergent, highly virulent strains (18). Structural reform may also be necessary as the criminalization of these three key populations is associated with worse HIV outcomes and inadequate viral suppression (37, 38), potentially accelerating HIV-1 diversification.

In summary, this is the first study to comprehensively analyse the global association of key populations with HIV-1 recombinants. PWID, CSW, and MSM were significantly associated with recombinants globally and across multiple regions. As key populations and their partners account for 70% of new HIV infections (1), it is apparent that key populations are driving the genetic diversification of the global HIV-1 pandemic, posing a challenge to diagnostics, treatments, and vaccines against HIV. Therefore, additional surveillance of HIV-1 molecular epidemiology and increased preventative measures should be targeted toward these key populations.

## Data availability statement

The original contributions presented in the study are included in the article/Supplementary material, further inquiries can be directed to the corresponding author.

## Ethics statement

Ethical review and approval was not required for the study on human participants in accordance with the local legislation and institutional requirements. Written informed consent for participation was not required for this study in accordance with the national legislation and the institutional requirements.

## Author contributions

NN assessed eligibility of manuscripts, conducted the analyses, designed figures and tables, interpreted the data, and wrote the manuscript. RE, JY, and LD-T screened the electronic literature search results for relevant manuscripts, assessed their eligibility, extracted data, and collected additional published data. SK designed and did the electronic literature search. JH conceived, designed, coordinated the study, wrote the systematic review protocol, assisted with the literature search, assessed eligibility of manuscripts, collected additional published data, conducted the global survey, extracted data, designed the analysis plan, interpreted the data, and wrote the manuscript. All authors read and approved the final version of the manuscript.

## WHO-UNAIDS Network for HIV Isolation and Characterisation

Alash'le G. Abimiku, Simon Agwale, Chris Archibald, Boaz Avidor, María Gabriela Barbás, Francoise Barre-Sinoussi, Banson Barugahare, El Hadj Belabbes, Silvia Bertagnolio, Deborah Birx, Aleksei F. Bobkov, James Brandful, Helba Bredell, Catherine A. Brennan, James Brooks, Marie Bruckova, Luigi Buonaguro, Franco Buonaguro, Stefano Buttò, Anne Buve, Mary Campbell, Jean Carr, Alex Carrera, Manuel Gómez Carrillo, Connie Celum, Beth Chaplin, Macarthur Charles, Dimitrios Chatzidimitriou, Zhiwei Chen, Katsumi Chijiwa, David Cooper, Philip Cunningham, Anoumou Dagnra, Cillian F. de Gascun, Julia Del Amo, Elena Delgado, Ursula Dietrich, Dominic Dwyer, Dennis Ellenberger, Barbara Ensoli, Max Essex, Herve Fleury, Peter N. Fonjungo, Vincent Foulongne, Deepak A. Gadkari, Feng Gao, Federico García, Roger Garsia, Guy Michel Gershy-Damet, Judith R. Glynn, Ruth Goodall, Zehava Grossman, Monick Lindenmeyer Guimarães, Beatrice Hahn, Raph L. Hamers, Osamah Hamouda, Ray Handema, Xiang He, Joshua Herbeck, David D. Ho, Africa Holguin, Mina Hosseinipour, Gillian Hunt, Masahiko Ito, Mohamed Ali Bel Hadj Kacem, Erin Kahle, Pontiano Kaleebu, Marcia Kalish, Adeeba Kamarulzaman, Chun Kang, Phyllis Kanki, Edward Karamov, Jean-Claude Karasi, Kayitesi Kayitenkore, Tony Kelleher, Dwip Kitayaporn, Leondios G. Kostrikis, Claudia Kucherer, Claudia Lara, Thomas Leitner, Kirsi Liitsola, Jai Lingappa, Marek Linka, Ivette Lorenzana de Rivera, Vladimir Lukashov, Shlomo Maayan, Luzia Mayr, Francine McCutchan, Nicolas Meda, Elisabeth Menu, Fred Mhalu, Doreen Mloka, John L. Mokili, Brigitte Montes, Orna Mor, Mariza Morgado, Fausta Mosha, Awatef Moussi, James Mullins, Rafael Najera, Mejda Nasr, Nicaise Ndembi, Joel R. Neilson, Vivek R. Nerurkar, Florian Neuhann, Claudine Nolte, Vlad Novitsky, Philippe Nyambi, Marianna Ofner, Fem J. Paladin, Anna Papa, Jean Pape, Neil Parkin, Chris Parry, Martine Peeters, Alexandra Pelletier, Lucía Pérez-Álvarez, Deenan Pillay, Angie Pinto, Trinh Duy Quang, Cecilia Rademeyer, Filimone Raikanikoda, Mark A. Rayfield, Jean-Marc Reynes, Tobias Rinke de Wit, Kenneth E. Robbins, Morgane Rolland, Christine Rousseau, Jesus Salazar-Gonzales, Hanan Salem, Mika Salminen, Horacio Salomon, Paul Sandstrom, Mario L. Santiago, Abdoulaye D. Sarr, Bryan Schroeder, Michel Segondy, Philippe Selhorst, Sylvester Sempala, Jean Servais, Ansari Shaik, Yiming Shao, Amine Slim, Marcelo A. Soares, Elijah Songok, Debbie Stewart, Julie Stokes, Shambavi Subbarao, Ruengpung Sutthent, Jun Takehisa, Amilcar Tanuri, Kok Keng Tee, Kiran Thapa, Michael Thomson, Tyna Tran, Willy Urassa, Hiroshi Ushijima, Philippe van de Perre, Guido van der Groen, Kristel van Laethem, Joep van Oosterhout, Ard van Sighem, Eric van Wijngaerden, Anne-Mieke Vandamme, Jurgen Vercauteren, Nicole Vidal, Lesley Wallace, Carolyn Williamson, Dawit Wolday, Jianqing Xu, Chunfu Yang, Linqi Zhang, Rong Zhang.

## Affiliations

Institute of Human Virology, University of Maryland, Baltimore, MD, USA (A. G. Abimiku, J. Carr); Gede Foundation, Abuja, Nigeria (S. Agwale); Public Health Agency of Canada, Ottawa, ON, Canada (C. Archibald, J. Brooks, M. Ofner, P. Sandstrom, J. Stokes); Tel-Aviv Sourasky Medical Center, Tel-Aviv, Israel (B. Avidor); Ministerio de Salud, Córdoba, Argentina (M. G. Barbás); Institut Pasteur, Paris, France (F. Barre-Sinoussi, E. Menu); Ministry of Health, Entebbe, Uganda (B. Barugahare); National Reference Laboratory on HIV/AIDS, Institut Pasteur d'Algérie, Algiers, Algeria (E. Belabbes); World Health Organization, Geneva, Switzerland (S. Bertagnolio); Office of the Global AIDS Coordinator, Washington, DC, USA (D. Birx); The D I Ivanovsky Institute of Virology, Moscow, Russia (A. F. Bobkov); Noguchi Memorial Institute for Medical Research, University of Ghana, Accra, Ghana (J. Brandful); University of Cape Town, Cape Town, South Africa (H. Bredell, C. Rademeyer, P. Selhorst, D. Stewart, C. Williamson); Abbott Laboratories, Chicago, IL, USA (C. A. Brennan); National Institute of Public Health, Prague, Czech Republic (M. Bruckova, M. Linka); AIDS Reference Center, National Cancer Institute “Fond. G. Pascale”, Naples, Italy (L. Buonaguro, F. Buonaguro); National AIDS Center, Istituto Superiore di Sanità, Rome, Italy (S. Buttò, B. Ensoli); Institute of Tropical Medicine, Antwerp, Belgium (A. Buve, G. van der Groen); University of Washington School of Medicine, Seattle, WA, USA (M. Campbell, C. Celum, J. Herbeck, E. Kahle, J. Lingappa, J. Mullins, M. Rolland, C. Rousseau); St Vincent's Hospital, Sydney, Australia (A. Carrera, P. Cunningham); University of Buenos Aires, Buenos Aires, Argentina (M. Carrillo, H. Salomon); Harvard T H Chan School of Public Health, Boston, MA, USA (B. Chaplin, P. Kanki); Gheskio Center, Port-au-Prince, Haiti (M. Charles, C. Nolte, J. Pape); Aristotle University of Thessaloniki, Thessaloniki, Greece (D. Chatzidimitriou, A. Papa); Chinese Academy of Medical Sciences, Peking Union Medical School, Beijing, China (Z. Chen, L. Zhang, R. Zhang); Fukuoka Institute of Health and Environmental Sciences, Kyushu University Hospital, Dazaifu, Japan (K. Chijiwa); The Kirby Institute, Sydney, NSW, Australia (D. Cooper, T. Kelleher, A. Pinto, A. Shaik); Faculté des Sciences de la Santé, Université de Lomé, Lomé, Togo (A. Dagnra); University College Dublin, Dublin, Ireland (C. de Gascun); Instituto de Salud Carlos III, Madrid, Spain (J. Del Amo, E. Delgado, R. Najera, L. Pérez-Álvarez, M. Thomson); Chemotherapeutisches Forschungsinstitut, Georg-Speyer-Haus, Frankfurt, Germany (U. Dietrich); Pathology West, Westmead Hospital, Westmead, NSW, Australia (D. Dwyer, K. Thapa, T. Tran); Centers for Disease Control and Prevention, Atlanta, GA, USA (D. Ellenberger, P. N. Fonjungo, M. A. Rayfield, K. E. Robbins, S. Subbarao, C. Yang); Harvard School of Public Health, Boston, MA, USA (M. Essex, V. Novitsky, A. D. Sarr); Duke University Medical Center, Durham, NC, USA (F. Gao); University of Bordeaux, Bordeaux, France (H. Fleury); Montpellier University Hospital, Montpellier, France (V. Foulongne, P. van de Perre); National AIDS Research Institute, Pune, India (D. A. Gadkari); Complejo Hospitalario Universitario de Granada, Granada, Spain (F. García); Royal Prince Alfred Hospital, Sydney, Australia (R. Garsia, H. Salem); HIV Laboratory Programme on AIDS/AFRO, World Health Organisation, Ouagadougou, Burkina Faso (G. M. Gershy-Damet); London School of Hygiene and Tropical Medicine, London, UK (J. R. Glynn); University College London, London, UK (R. Goodall); National HIV Reference Laboratory, Ministry of Health, Tel Aviv, Israel (Z. Grossman, O. Mor); Instituto Oswaldo Cruz, FIOCRUZ, Rio de Janeiro, Brazil (M. L. Guimarães, M. Morgado); University of Pennsylvania, Philadelphia, PN, USA (B. Hahn); Amsterdam Institute for Global Health and Development, Amsterdam, Netherlands (R. L. Hamers, T. Rinke de Wit); Robert Koch Institute, Berlin, Germany (O. Hamouda, C. Kucherer); Yamanashi Medical University, Yamanashi, Japan (R. Handema, M. Ito); National Center for AIDS/STD Control and Prevention, China CDC, Beijing, China (X. He, Y. Shao, J. Xu); Aaron Diamond AIDS Research Center, The Rockefeller University, New York, NY, USA (D. D. Ho, L. G. Kostrikis); Ramón y Cajal Research Institute, Hospital Universitario Ramón y Cajal de Madrid, Madrid, Spain (A. Holguin); University of North Carolina, Chapel Hill, NC, USA (M. Hosseinipour); National Institute for Communicable Diseases, Johannesburg, South Africa (G. Hunt); Charles Nicolle Hospital, Tunis, Tunisia (M. Kacem, A. Moussi, M. Nasr, A. Slim); Medical Research Council, Entebbe, Uganda (P. Kaleebu, C. Parry); Vanderbilt Institute for Global Health, Vanderbilt University School of Medicine, Nashville, TN, USA (M. Kalish); University of Malaya, Kuala Lumpur, Malaysia (A. Kamarulzaman, K-K Tee); Institute for Molecular Biology and Genetics and Medical College, Seoul National University, Seoul, Korea (C. Kang); Gamaleya Center for Epidemiology and Microbiology, Moscow, Russian (E. Karamov); National Reference Laboratory, Kigali, Rwanda (J-C Karasi); Emory University School of Medicine, Atlanta, GA, USA (K. Kayitenkore); HIV/AIDS Collaboration, Nonthaburi, Thailand (D. Kitayaporn); Karolinska Institute, Huddinge University Hospital, Stockholm, Sweden (C. Lara); Los Alamos National Laboratory, Los Alamos, NM, USA (T. Leitner); National Institute for Health and Wellfare, Helsinki, Finland (K. Liitsola, M. Salminen); National Autonomous University of Honduras, Tegucigalpa, Honduras (I. Lorenzana de Rivera); Academic Medical Center, University of Amsterdam, Amsterdam, Netherlands (V. Lukashov); Hadassah U Hospital, Jerusalem, Israel (S. Maayan); New York University School of Medicine, New York, NY, USA (L. Mayr, P. Nyambi); Henry M. Jackson Foundation for the Advancement of Military Medicine, Bethesda, MD, USA (F. McCutchan); Centre Muraz, Bobo-Dioulasso, Burkina Faso (N. Meda); Muhimbili University of Health Sciences, Dar-es-salaam, Tanzania (F. Mhalu, D. Mloka, F. Mosha, W. Urassa); University of Edinburgh, Edinburgh, UK (J. L. Mokili); Montpellier University Hospital, Montpellier, France (B. Montes, M. Segondy); Institute of Human Virology, Abuja, Nigeria (N. Ndembi); University of Washington, Seattle, WA, USA (J. R. Neilson); University of Hawaii, Honolulu, HI, USA (V. R. Nerurkar); University Clinic Heidelberg, Heidelberg, Germany & Lighthouse Trust, Lilongwe, Malawi (F. Neuhann); Research Institute for Tropical Medicine, Muntinlupa City, Manila, Philippines (F. J. Paladin, M. L. Santiago); Data First Consulting, Inc, Belmont, CA, USA (N. Parkin); University of Montpellier, Montpellier, France (M. Peeters, N. Vidal); Centre de Recherche Public-Santé, Luxembourg, Luxembourg (A. Pelletier, J. Servais); Africa Health Research Institute, Durban, KwaZulu-Natal, South Africa & Division of Infection and Immunity, University College London, London, UK (D. Pillay); Institute of International Health, University of Tokyo, Tokyo, Japan (T. D. Quang); University of Sydney, Sydney, NSW, Australia (F. Raikanikoda); Institut Pasteur du Cambodge, Phnom Penh, Cambodia (J-M Reynes); University of Alabama at Birmingham, Birmingham, AL, USA (J. Salazar-Gonzales); Auckland City Hospital, Auckland, New Zealand (B. Schroeder); Uganda Virus Research Institute, Entebbe, Uganda (S. Sempala); Instituto Nacional de Câncer, Rio de Janeiro, Brazil (M. A. Soares); Kenya Medical Research Institute, Nairobi, Kenya (E. Songok); National HIV Repository and Bioinformatic Center, Siriraj Hospital, Mahidol University, Thailand (R. Sutthent); Laboratory of Viral Pathogenesis, Kyoto University, Kyoto, Japan (J. Takehisa); Federal University of Rio de Janeiro, Rio de Janeiro, Brazil (A. Tanuri); Aino Health Science Center and Aino University, Tokyo, Japan (H. Ushijima); Rega Institute for Medical Research, KU Leuven, Belgium (K. van Laethem, E. van Wijngaerden, A.-M. Vandamme, J. Vercauteren); Department of Medicine, Blantyre, Malawi (J. van Oosterhout); Stichting HIV Monitoring, Amsterdam, Netherlands (A. van Sighem); Health Protection Scotland, Glasgow, UK (L. Wallace); and Ethiopian Health & Nutrition Research Institute, Addis Ababa, Ethiopia (D. Wolday).
