# Supplementary material for: Global associations of key populations with HIV-1 recombinants: a systematic review, global survey, and individual participant data meta-analysis
Source: Front Public Health. 2023 Jul 27;11:1153638. doi: 10.3389/fpubh.2023.1153638 (PMC10420084; doi:10.3389/fpubh.2023.1153638)
Supplement: Supplementary file 1 [file Data_Sheet_1.docx]

**Global associations between key populations and HIV-1 recombinants: a systematic review, global survey, and individual participant data meta-analysis.**

Appendices

[Appendix 1: Search Strategies for literature databases. 3](#_Toc116845591)

[Appendix 2: Additional published data sources. 7](#_Toc116845592)

[Appendix 3: Countries per region. 10](#_Toc116845593)

[Appendix 4: Data collection on key populations and HIV-1 variants across regions, 1990-2015. 11](#_Toc116845594)

[Appendix 5: Global associations of key populations and HIV-1 recombinants, 1990-2015. 12](#_Toc116845595)

[Appendix 6: Regional associations of key populations and HIV-1 recombinants, 1990-2015. 13](#_Toc116845596)

[Appendix 7: Global associations of HIV-1 CRFs and URFs for key populations, relative to heterosexual people, for each time period. 15](#_Toc116845607)

[Appendix 8: Global associations of HIV-1 recombinants, CRFs, and URFs for key populations, relative to heterosexual people, for each time period. 16](#_Toc116845608)

[Appendix 9: Global distribution of HIV-1 subtypes, individual CRFs, and URFs among key populations, 1990-2015. 17](#_Toc116845609)

[Appendix 10: Regional distribution of HIV-1 subtypes, CRFs, and URFs among key populations, 1990-2015. 18](#_Toc116845610)

## Appendix 1: Search Strategies for literature databases.

**Pubmed** (via https://www.ncbi.nlm.nih.gov/pubmed/)

Search run in February 2015, update search run in February 2016:
1. subtyp* [tiab] OR subsubtype* [tiab] OR sub-subtype* [tiab] OR intrasubtype* [tiab] OR intra-subtype* [tiab] OR intersubtype* [tiab] OR inter-subtype* [tiab]
2. clade* [tiab] OR serotyp* [tiab] OR phylogen* [tiab] OR second generation recombinant* [tiab] OR second- generation recombinant* [tiab] OR SGR [tiab]
3. "circulating recombinant form" [tiab] OR CRF* [tiab] OR "unique recombinant form" [tiab] OR URF* [tiab] OR recombinant* [tiab] OR recombination* [tiab]
4. "molecular epidemiology" [tiab] OR "genetic diversity" [tiab] OR "genetic variation" [tiab] OR genetic variabilit* [tiab] OR mosaic* [tiab] OR "molecular diversity" [tiab]
5. Serotyping [mh] Phylogeny [mh] OR Recombination, Genetic [mh] OR Homologous Recombination [mh] OR Molecular Epidemiology [mh] OR Genetic Variation [mh] OR Mosaic Viruses [mh]
6. #1 OR #2 OR #3 OR #4 OR #5
7. HIV/genetics [mh] OR HIV Reverse Transcriptase/genetics [mh] OR HIV Integrase/genetics [mh] OR HIV Envelope Protein gp160/genetics [mh]
8. HIV Core Protein p24/genetics [mh] OR HIV Protease/genetics [mh] OR HIV Long Terminal Repeat/genetics [mh] OR HIV Enhancer/genetics [mh] OR HIV Envelope Protein gp41/genetics [mh] OR HIV Envelope Protein gp120/genetics [mh]
9. HIV Infections/genetics [mh] OR HIV Seroprevalence [mh] OR HIV-1/genetics [mh] OR HIV Antigens/genetics [mh] OR HIV Seropositivity/genetics [mh] OR rev Gene Products, Human Immunodeficiency Virus/genetics [mh]
10. tat Gene Products, Human Immunodeficiency Virus/genetics [mh] OR nef Gene Products, Human Immunodeficiency Virus/genetics [mh] OR AIDS Serodiagnosis [mh]
11. vpr Gene Products, Human Immunodeficiency Virus/genetics [mh] OR vif Gene Products, Human Immunodeficiency Virus/genetics [mh] OR Human Immunodeficiency Virus Proteins/genetics [mh]
12. pol Gene Products, Human Immunodeficiency Virus/genetics [mh] OR gag Gene Products, Human Immunodeficiency Virus/genetics [mh] OR env Gene Products, Human Immunodeficiency Virus/genetics [mh] 13. HIV [tiab] OR HIV-1 [tiab] OR HIV-type-1 [tiab] OR HIV-positive [tiab] OR HIV-1-positive [tiab] OR HIV- infected [tiab] OR HIV-1-infected [tiab] OR HIV-type-1-infected [tiab]
14. HIV-infection* [tiab] OR HIV-1-infection* [tiab] OR HIV-type-1-infection* [tiab] OR Human Immunodeficiency Virus* [tiab] OR "Human Immune Deficiency Virus*" [tiab] OR AIDS [tiab] OR ìAcquired Immune Deficiency Syndromeî [tiab] OR ìAcquired Immunodeficiency Syndromeî [tiab]
15. #7 OR #8 OR #9 OR #10 OR #11 OR #12 OR #13 OR #14
16. animals [mh] NOT (humans [mh] AND animals [mh])
17. #6 AND #15
18. #17 NOT #16
19. 1990:2015 [dp]
20. #18 AND #19

**Embase** (via Ovid)

Search run in February 2015, update search run in February 2016:
1. (subtyp$ OR subsubtype$ OR sub-subtype$ OR intrasubtype$ OR intra-subtype$ OR intersubtype$ OR inter- subtype$).ti,ab.
2. (clade$ OR serotyp$ OR phylogen$ OR SGR).ti,ab.
3. (second adj generation adj recombinant$).ti,ab.
4. (second-generation adj recombinant$).ti,ab.

5. (circulating adj recombinant adj form).ti,ab.
6. (CRF$ OR URF$ OR recombination$ OR recombinant$ OR mosaic$).ti,ab.
7. (unique adj recombinant adj form).ti,ab.
8. (molecular adj epidemiology).ti,ab.
9. (genetic adj diversity).ti,ab.
10. (genetic adj variation).ti,ab.
11. (genetic adj variabilit$).ti,ab.
12. (molecular adj diversity).ti,ab.
13. Serotype/
14. Serotyping/
15. Phylogeny/
16. Molecular Phylogeny/
17. Genetic Recombination/
18. Homologous Recombination/
19. Molecular Epidemiology/
20. Genetic Variability/
21. Virus Recombination/
22. Mosaicism/
23. OR/1-22
24. Human immunodeficiency virus/
25. Human immunodeficiency virus 1/
26. Human immunodeficiency virus 1 infection/
27. Acquired immune deficiency syndrome/
28. Human immunodeficiency virus infection/
29. Human immunodeficiency virus prevalence/
30. Human immunodeficiency virus antigen/
31. Human immunodeficiency virus proteinase/
32. Human Immunodeficiency Virus Protein/
33. (HIV OR HIV-1 OR HIV-type-1 OR HIV-positive OR HIV-1-positive OR HIV-infected OR HIV-1-infected OR HIV-type-1-infected).ti,ab.
34. (HIV-infection$ OR HIV-1-infection$ OR HIV-type-1-infection$ OR AIDS).ti,ab.
35. (Human adj Immunodeficiency adj Virus$).ti,ab.
36. (Human adj Immune adj Deficiency adj Virus$).ti,ab.
37. (Acquired adj Immune adj Deficiency adj Syndrome).ti,ab.
38. (Acquired adj Immunodeficiency adj Syndrome).ti,ab.
39. OR/24-38
40. animal/
41. human/
42. 40 and 41
43. 40 not 42
44. 23 and 39
45. 44 not 43
46. Limit 45 to yr=1990-2015

**CINAHL** (via EbscoHost)

Search run in February 2015, update search run in February 2016: 1. (TI subtyp* OR AB subtyp*)
2. (TI subsubtype* OR AB subsubtype*)
3. (TI sub-subtype* OR AB sub-subtype*)

4. (TI intrasubtype* OR AB intrasubtype*)

5. (TI intra-subtype* OR AB intra-subtype*)

6. (TI intersubtype* OR AB intersubtype*)
7. (TI inter-subtype* OR AB inter-subtype*)
8. (TI clade* OR AB clade*)
9. (TI serotyp* OR AB serotyp*)
10. (TI phylogen* OR AB phylogen*)
11. (TI "second generation recombinant*" OR AB "second generation recombinant*")
12. (TI SGR OR AB SGR)
13. (TI "circulating recombinant form" OR AB "circulating recombinant form")
14. (TI CRF* OR AB CRF*)
15. (TI "unique recombinant form" OR AB "unique recombinant form")
16. (TI URF* OR AB URF*)
17. (TI recombinant* OR AB recombinant*)
18. (TI recombination* OR AB recombination*)
19. (TI "molecular epidemiology" OR AB "molecular epidemiology")
20. (TI "genetic diversity" OR AB "genetic diversity")
21. (TI "genetic variation" OR AB "genetic variation")
22. (TI genetic variabilit* OR AB genetic variabilit*)
23. (TI mosaic* OR AB mosaic*)
24. (TI "molecular diversity" OR AB "molecular diversity")
25. (MH Serotyping OR MJ Serotyping OR MW Serotyping)
26. (MH "Epidemiology, Molecular" OR MJ "Epidemiology, Molecular" OR MW "Epidemiology, Molecular") 27. S1 OR S2 OR S3 OR S4 OR S5 OR S6 OR S7 OR S8 OR S9 OR S10 OR S11 OR S12 OR S13 OR S14 OR S15 OR S16 OR S17 OR S18 OR S19 OR S20 OR S21 OR S22 OR S23 OR S24 OR S25 OR S26
28. (MH "Human Immunodeficiency Virus" OR MJ "Human Immunodeficiency Virus" OR MW "Human Immunodeficiency Virus")
29. (MH "HIV-Infected Patients" OR MJ "HIV-Infected Patients" OR MW "HIV-Infected Patients")
30. (MH "HIV Infections" OR MJ "HIV Infections" OR MW "HIV Infections")
31. (MH HIV-1 OR MJ HIV-1 OR MW HIV-1)
32. (MH "HIV Seropositivity" OR MJ "HIV Seropositivity" OR MW "HIV Seropositivity")
33. (MH "HIV Seronegativity" OR MJ "HIV Seronegativity" OR MW "HIV Seronegativity")
34. (MH "AIDS Serodiagnosis" OR MJ "AIDS Serodiagnosis" OR MW "AIDS Serodiagnosis")
35. (TI HIV OR AB HIV)
36. (TI HIV-1 OR AB HIV-1)
37. (TI HIV-type-1 OR AB HIV-type-1)
38. (TI HIV-positive OR AB HIV-positive)
39. (TI HIV-1-positive OR AB HIV-1-positive)
40. (TI HIV-infected OR AB HIV-infected)
41. (TI HIV-1-infected OR AB HIV-1-infected)
42. (TI HIV-type-1-infected OR AB HIV-type-1-infected)
43. (TI HIV-infection* OR AB HIV-infection*)
44. (TI HIV-1-infection* OR AB HIV-1-infection*)
45. (TI HIV-type-1-infection* OR AB HIV-type-1-infection*)
46. (TI "Human Immunodeficiency Virus*" OR AB "Human Immunodeficiency Virus*")
47. (TI "Human Immune Deficiency Virus*" OR AB "Human Immune Deficiency Virus*")
48. (TI AIDS OR AB AIDS)
49. (TI Acquired Immune Deficiency Syndrome OR AB Acquired Immune Deficiency Syndrome)
50. (TI Acquired Immunodeficiency Syndrome OR AB Acquired Immunodeficiency Syndrome)
51. S28 OR S29 OR S30 OR S31 OR S32 OR S33 OR S34 OR S35 OR S36 OR S37 OR S38 OR S39 OR S40 OR S41 OR S42 OR S43 OR S44 OR S45 OR S46 OR S47 OR S48 OR S49 OR S50
52. S27 AND S51
53. PY 1990-2015
54. S52 AND S53

**Global Health** (via Ovid)

Search run in February 2015, update search run in February 2016:
1. (subtyp$ OR subsubtype$ OR sub-subtype$ OR intrasubtype$ OR intra-subtype$ OR intersubtype$ OR inter- subtype$).ti,ab.
2. (clade$ OR serotyp$ OR phylogen$ OR SGR).ti,ab.
3. (second adj generation adj recombinant$).ti,ab.
4. (second-generation adj recombinant$).ti,ab.
5. (circulating adj recombinant adj form).ti,ab.
6. (CRF$ OR URF$ OR recombination$ OR recombinant$ OR mosaic$).ti,ab.
7. (unique adj recombinant adj form).ti,ab.
8. (molecular adj epidemiology).ti,ab.
9. (genetic adj diversity).ti,ab.
10. (genetic adj variation).ti,ab.
11. (genetic adj variabilit$).ti,ab.
12. (molecular adj diversity).ti,ab.
13. Serotypes/
14. Phylogeny/
15. Recombination/
16. Homologous Recombination/
17. Molecular Epidemiology/
18. Genetic Variation/
19. Mosaicism/
20. Phylogenetics/
21. Genetic variance/
22. OR/1-21
23. Human immunodeficiency viruses/
24. HIV infections/
25. Human immunodeficiency virus 1/
26. HIV-1 infections/
27. Acquired immune deficiency syndrome/
28. (HIV OR HIV-1 OR HIV-type-1 OR HIV-positive OR HIV-1-positive OR HIV-infected OR HIV-1-infected OR HIV-type-1-infected).ti,ab.
29. (HIV-infection$ OR HIV-1-infection$ OR HIV-type-1-infection$ OR AIDS).ti,ab.
30. (Human adj Immunodeficiency adj Virus$).ti,ab.
31. (Human adj Immune adj Deficiency adj Virus$).ti,ab.
32. (Acquired adj Immune adj Deficiency adj Syndrome).ti,ab.
33. (Acquired adj Immunodeficiency adj Syndrome).ti,ab.
34. OR/23-33
35. 22 and 34
36. Limit 35 to yr=1990-2015

## Appendix 2: Additional published data sources.

Published data sources which contained references of published studies with primary data not found through the electronic literature search:

**i)** WHO HIV Drug Resistance Report, World Health Organization, Geneva, 2012. Annex 2, Table 12.

**ii)** Published reports and reviews on HIV diversity:

Ariën KK, Vanham G, Arts EJ. Is HIV-1 evolving to a less virulent form in humans? *Nature Reviews Microbiology* 2007; 5(2): 141-151.

Bao YP, et al. Review of HIV and HCV infection among drug users in China. *Current Opinion in Psychiatry* 2010; 23(3): 187-194.

Barth RE, et al. Virological follow-up of adult patients in antiretroviral treatment programmes in sub-Saharan Africa: a systematic review. *Lancet Infectious Diseases* 2010; 10(3): 155-166.

Bhargava M, et al. Do HIV-1 non-B subtypes differentially impact resistance mutations and clinical disease progression in treated populations? Evidence from a systematic review. *Journal of the International AIDS Society* 2014; 17(18944).

Brindicci G, et al. HIV-1 nonsubtype B distribution across certain resource-rich countries. *HIV Therapy* 2009; 3(5): 467-483.

Buonaguro L, et al. Human immunodeficiency virus type 1 subtype distribution in the worldwide epidemic: pathogenetic and therapeutic implications. *J Virology* 2007; 81(19): 10209-19.

Chan PA, et al. Short communication: New HIV infections at southern new england academic institutions: Implications for prevention. *AIDS Research and Human Retroviruses* 2013; 29(1): 25-29.

Choy KK. A review of HIV/AIDS research in Malaysia. *Medical Journal of Malaysia* 2014; 69: 68-81.

Garber DA, Silvestri G, Feinberg MB. Prospects for an AIDS vaccine: three big questions, no easy answers. *Lancet Infect Dis* 2004; 4(7): 397-413.

Geretti AM. HIV-1 subtypes: epidemiology and significance for HIV management. *Curr Opin Infect Dis* 2006; 19(1): 1-7.

Graf T, Pinto AR. The increasing prevalence of HIV-1 subtype C in Southern Brazil and its dispersion through the continent. *Virology* 2013; 435(1): 170-178.

Holguin A, et al. Increase of non-B subtypes and recombinants among newly diagnosed HIV-1 native spaniards and immigrants in Spain. *Current HIV Research* 2008; 6(4): 327-334.

Janssen S, et al. TB and HIV in the Central African region: Current knowledge and knowledge gaps. *Infection* 2014; 42(2): 281-294.

Kalish ML, Robbins KE, Pieniazek D, et al. Recombinant viruses and early global HIV-1 epidemic. *Emerging Infect Dis* 2004; 10(7): 1227-34.

Kantor R, et al. Impact of HIV-1 pol diversity on drug resistance and its clinical implications. *Curr Opin Infect Dis* 2006; 19(6): 594-606.

Kouyoumjian SP, et al. The epidemiology of HIV infection in Morocco: Systematic review and data synthesis. *International Journal of STD and AIDS* 2013; 24(7): 507-516.

Lihana R, et al. Update on HIV-1 diversity in Africa: a decade in review. *AIDS Rev* 2012; 14(2): 83-100.

Luft LM, et al. HIV-1 viral diversity and its implications for viral load testing: Review of current platforms. *International Journal of Infectious Diseases* 2011; 15(10): e661-e670.

Manolescu LS, et al. HIV-1 circulating subtypes in Romania. *Roumanian Archives of Microbiology and Immunology* 2013; 72(2): 121-134.

Martinez-Cajas J L, et al. Role of genetic diversity amongst HIV-1 non-B subtypes in drug resistance: A systematic review of virologic and biochemical evidence. *AIDS Reviews* 2008; 10(4): 212-223.

McCutchan F. Global epidemiology of HIV. J Med Virol. 2006; 78 Suppl 1: S7-S12.

Meng Z, et al. A New Migration Map of HIV-1 CRF07_BC in China: Analysis of Sequences from 12 Provinces over a Decade. *PLoS ONE* 2012; 7(12).

Merati TP, et al. CRF01-AE dominates the HIV-1 epidemic in Indonesia. *Sexual Health* 2012; 9(5): 414-421.

Mumtaz G, et al. HIV-1 molecular epidemiology evidence and transmission patterns in the Middle East and North Africa. *Sex Transm Infect* 2011; 87(2): 101-6.

Nájera R, et al. Genetic recombination and its role in the development of the HIV-1 pandemic. *AIDS* 2002; 16 Suppl 4: S3-16.

Neogi U, et al. Global HIV-1 molecular epidemiology with special reference to genetic analysis of HIV-1 subtypes circulating in North India: functional and pathogenic implications of genetic variation. *Indian Journal of Experimental Biology* 2009; 47(6): 424-431.

Neogi U, et al. Genetic architecture of HIV-1 genes circulating in north India & their functional implications. *Indian Journal of Medical Research* 2011; 134(12): 769-778.

Ng OT, et al. Molecular epidemiology of HIV type 1 in Singapore and identification of novel CRF01-AE/B recombinant forms. *AIDS Research and Human Retroviruses* 2011 27(10): 1135-1137.

Obiako OR, et al. Antiretroviral drug resistance-implications for HIV/AIDS reduction in sub-Saharan Africa and other developing countries. *Nigerian Journal of Medicine* 2010 19(4): 352-360.

Ouyang Y, et al. HIV-1 CRF_BC recombinants infection in China: Molecular epidemic and characterizations. *Current HIV Research* 2012; 10(2): 151-161.

Papathanasopoulos M, et al. Evolution and diversity of HIV-1 in Africa-a review. *Virus Genes* 2003; 26(2): 151- 63.

Parczewski M. Subtype variability and phylogenetic analyses in HIV. *HIV and AIDS Review* 2013; 12(4): 93-96.

Raboni SM, et al. Molecular epidemiology of HIV-1 clades in Southern Brazil. *Memorias do Instituto Oswaldo Cruz* 2010; 105(8): 1044-1049.

Rhee S-Y, et al. Geographic and Temporal Trends in the Molecular Epidemiology and Genetic Mechanisms of Transmitted HIV-1 Drug Resistance: An Individual-Patient- and Sequence- Level Meta-Analysis. *PLoS Medicine* 2015; 12(4): e1001810.

Santos AF, Soares MA. HIV genetic diversity and drug resistance. *Viruses* 2010; 2(2): 503-531.

Sendagire H, et al. The challenge of HIV-1 antiretroviral resistance in Africa in the era of HAART. *AIDS Reviews* 2009; 11(2): 59-70.

Singh K, et al. Drug resistance in non-B subtype HIV-1: Impact of HIV-1 reverse transcriptase inhibitors. *Viruses* 2014; 6(9): 3535-3562.

Soares MA. Drug resistance differences among HIV types and subtypes: A growing problem. *Future HIV Therapy* 2008; 2(6): 579-593.

Stanojevic M, et al. HIV-1 molecular epidemiology in the Balkans: a melting pot for high genetic diversity. *AIDS Rev* 2012; 14(1): 28-36.

Taiwo B, et al. Novel antiretroviral combinations in treatment-experienced patients with HIV infection: Rationale and results. *Drugs* 2010; 70(13): 1629-1642.

Takebe Y, et al. Molecular epidemiology of HIV: tracking AIDS pandemic. *Pediatr Int* 2004; 46(2): 236-44.

Tebit D, et al. HIV diversity, recombination and disease progression: how does fitness "fit" into the puzzle? *AIDS Rev* 2007; 9(2): 75-87.

Thomson M, et al. Molecular epidemiology of HIV-1 genetic forms and its significance for vaccine development and therapy. *Lancet Infect Dis* 2002; 2(8): 461-71.

Thomson MM, Nájera R. Molecular epidemiology of HIV-1 variants in the global AIDS pandemic: an update. *AIDS Rev* 2005; 7(4): 210-24.

Woodman Z, Williamson C. HIV molecular epidemiology: Transmission and adaptation to human populations. *Current Opinion in HIV and AIDS* 2009; 4(4): 247-252.

World Health Organization, WHO HIV Drug Resistance Report, Geneva, 2012. Annex 1, Table 1.

Zhang L, et al. Prevalence of HIV-1 subtypes among men who have sex with men in China: a systematic review. *Int J STD AIDS* 2015; 26(5): 291-305.

**iii)** Tables of Content of four specialist journals (AIDS, Journal of AIDS, Journal of Virology, and AIDS Research and Human Retroviruses) of issues published between January 1990 and February 2016.

**iv)** Scopus citation database1(last accessed 1 February 2016): search identified papers which referenced previous publications on global HIV-1 molecular epidemiology.2-4

1. Scopus database of peer-reviewed literature. https://www.scopus.com/ (last accessed 1 February 2016).
2. Hemelaar J. et al. Global trends in molecular epidemiology of HIV-1 during 2000-2007. *AIDS* 2011; 25(5): 679-89.
3. Hemelaar J, et al. Global and regional distribution of HIV-1 genetic subtypes and recombinants in 2004. *AIDS* 2006; 20(16): W13-23.
4. Osmanov S, et al. Estimated global distribution and regional spread of HIV-1 genetic subtypes in the year 2000. *JAIDS* 2002; 29:184-90.

## Appendix 3: Countries per region.

**Caribbean:** Bahamas, Barbados, Cuba, Dominican Republic, Haiti, Jamaica, Trinidad and Tobago.
**Latin America:** Argentina**,** Belize**,** Bolivia, Brazil, Chile, Colombia, Costa Rica, Ecuador, El Salvador, Guatemala, Guyana, Honduras, Mexico, Nicaragua, Panama, Paraguay, Peru, Suriname, Uruguay, Venezuela. **Western and Central Europe and North America (WCENA):** Austria, Belgium, Bulgaria, Canada, Croatia, Cyprus, Czech Republic, Denmark, Estonia, Finland, France, Germany, Greece, Hungary, Iceland, Ireland, Israel, Italy, Latvia, Lithuania, Luxembourg, Malta, Netherlands, Norway, Poland, Portugal, Romania, Serbia, Slovakia, Slovenia, Spain, Sweden, Switzerland, Turkey, United Kingdom of Great Britain & Northern Ireland, United States of America.
**Eastern Europe & Central Asia (EECA):** Albania, Armenia, Azerbaijan, Belarus, Bosnia and Herzegovina, Georgia, Kazakstan, Kyrgyzstan, Montenegro, Republic of Moldova, Russian Federation, Tajikistan, The former Yugoslav Republic of Macedonia, Ukraine, Uzbekistan.
**South Asia:** India
**South-East Asia:** Afghanistan, Bangladesh, Bhutan, Brunei Darussalam, Cambodia, Indonesia, Lao People's Democratic Republic, Malaysia, Maldives, Myanmar, Nepal, Pakistan, Philippines, Singapore, Sri Lanka, Thailand, Timor-Leste, Vietnam.
**East Asia:** China, Democratic People's Republic of Korea, Japan, Mongolia, Republic of Korea.
**Oceania:** Australia, Fiji, New Zealand, Papua New Guinea.
**Middle East & North Africa:** Algeria, Egypt, Iran (Islamic Republic of), Lebanon, Morocco, Oman, Sudan, Tunisia, Yemen.
**West Africa:** Benin, Burkina Faso, Cameroon, Cape Verde, Cote d'Ivoire, Gambia, Ghana, Guinea, Guinea- Bissau, Liberia, Mali, Mauritania, Niger, Nigeria, Senegal, Sierra Leone, Togo.
**East Africa:** Burundi, Djibouti, Eritrea, Kenya, Madagascar, Mauritius, Rwanda, Somalia, South Sudan, Uganda, United Republic of Tanzania.
**Ethiopia:** Ethiopia.
**Central Africa:** Angola, Central African Republic, Chad, Congo, Democratic Republic of the Congo, Equatorial Guinea, Gabon.
**Southern Africa:** Botswana, Lesotho, Malawi, Mozambique, Namibia, South Africa, Swaziland, Zambia, Zimbabwe.

## Appendix 4: Data collection on key populations and HIV-1 variants across regions, 1990-2015.

|  | **Caribbean** | **Latin America** | **Western and central Europe, and North America** | **Eastern Europe and central Asia** | **South Asia** | **Southeast Asia** | **East Asia** | **Oceania** | **Middle East and North Africa** | **West Africa** | **East Africa** | **Ethiopia** | **Central Africa** | **Southern Africa** | **Total** |
| --- | --- | --- | --- | --- | --- | --- | --- | --- | --- | --- | --- | --- | --- | --- | --- |
| **Number of Datasets** | | | | | | | | | | | | | | | |
| 1990-2015 | 13  (1.5%) | 45  (5.1%) | 238  (26.9%) | 22  (2.5%) | 13  (1.5%) | 56  (6.3%) | 32  (3.6%) | 10  (1.1%) | 2  (0.2%) | 152  (17.2%) | 109  (12.3%) | 7  (0.8%) | 41  (4.6%) | 145  (16.4%) | 885  (100%) |
| 1990-1999 | 1 (0.5%) | 8 (4.1%) | 54 (28.0%) | 14 (7.3%) | 3 (1.6%) | 30 (15.5%) | 3 (1.6%) | 0 (0.0%) | 0 (0.0%) | 32 (16.6%) | 21 (10.9%) | 1 (0.5%) | 8 (4.1%) | 18 (9.3%) | 193 (100%) |
| 2000-2004 | 2 (1.0%) | 21 (11.0%) | 57 (29.8%) | 3 (1.6%) | 5 (2.6%) | 9 (4.7%) | 7 (3.7%) | 5 (2.6%) | 1 (0.5%) | 25 (13.1%) | 24 (12.6%) | 4 (2.1%) | 1 (0.5%) | 27 (14.1%) | 191 (100%) |
| 2005-2009 | 8 (2.5%) | 9 (2.9%) | 55 (17.5%) | 1 (0.3%) | 3 (1.0%) | 10 (3.2%) | 14 (4.4%) | 5 (1.6%) | 0 (0.0%) | 52 (16.5%) | 52 (16.5%) | 2 (0.6%) | 26 (8.3%) | 78 (24.8%) | 315 (100%) |
| 2010-2015 | 1 (0.6%) | 5 (2.9%) | 72 (41.1%) | 4 (2.3%) | 2 (1.1%) | 6 (3.4%) | 8 (4.6%) | 0 (0.0%) | 1 (0.6%) | 41 (23.4%) | 10 (5.7%) | 0 (0.0%) | 5 (2.9%) | 20 (11.4%) | 175 (100%) |
|  |  |  |  |  |  |  |  |  |  |  |  |  |  |  |  |
| **Number of Participants** | | | | | | | | | | | | | | | |
| 1990-2015 | 1,083  (1.4%) | 5,219  (6.8%) | 20,386  (26.4%) | 1,040  (1.3%) | 735  (1.0%) | 6,745  (8.7%) | 7,386  (9.6%) | 919  (1.2%) | 111  (0.1%) | 10,194  (13.2%) | 10,964  (14.2%) | 230  (0.3%) | 2,859  (3.7%) | 9,413  (12.2%) | 77, 284  (100%) |
| 1990-1999 | 27 (0.2%) | 379 (2.7%) | 1,875 (13.3%) | 676 (4.8%) | 104 (0.7%) | 2,322 (16.5%) | 161 (1.1%) | 0 (0.0%) | 0 (0.0%) | 2,332 (16.5%) | 4,070 (28.9%) | 40 (0.3%) | 824 (5.8%) | 1,289 (9.1%) | 14,099 (100%) |
| 2000-2004 | 133 (0.8%) | 1,510 (9.3%) | 5,985 (36.7%) | 126 (0.8%) | 414 (2.5%) | 1,461 (9.0%) | 413 (2.5%) | 192 (1.2%) | 71 (0.4%) | 1,888 (11.6%) | 2,425 (14.9%) | 115 (0.7%) | 31 (0.2%) | 1,538 (9.4%) | 16,302 (100%) |
| 2005-2009 | 654 (2.9%) | 975 (4.4%) | 5,788 (26.0%) | 41 (0.2%) | 113 (0.5%) | 1,185 (5.3%) | 2,506 (11.2%) | 727 (3.3%) | 0 (0.0%) | 2,219 (10.0%) | 3,203 (14.4%) | 75 (0.3%) | 1,365 (6.1%) | 3,430 (15.4%) | 22,281 (100%) |
| 2010-2015 | 127 (0.5%) | 2,288 (9.9%) | 6,738 (29.0%) | 197 (0.8%) | 104 (0.4%) | 1,580 (6.8%) | 4,306 (18.6%) | 0 (0.0%) | 40 (0.2%) | 3,394 (14.6%) | 990 (4.3%) | 0 (0.0%) | 532 (2.3%) | 2,916 (12.6%) | 23,212 (100%) |

## Appendix 5: Global associations of key populations and HIV-1 recombinants, 1990-2015.

| **HET** | 1.03 (1.00-1.07) | **0.38 (0.36-0.41)** | **1.73 (1.60-1.87)** | **0.63 (0.57-0.70)** | **2.32 (1.80-3.00)** |  |
| --- | --- | --- | --- | --- | --- | --- |
| 0.97 (0.93-1.01) | **MSM** | **0.37 (0.35-0.40)** | **1.67 (1.54-1.82)** | **0.61 (0.55-0.68)** | **2.25 (1.74-2.91)** |  |
| **2.60 (2.46-2.74)** | **2.68 (2.53-2.84)** | **PWID** | **4.48 (4.09-4.91)** | **1.64 (1.47-1.83)** | **6.03 (4.65-7.82)** |  |
| **0.58 (0.54-0.63)** | **0.60 (0.55-0.65)** | **0.22 (0.20-0.24)** | **VERT** | **0.37 (0.32-0.41)** | **1.34 (1.03-1.75)** |  |
| **1.59 (1.44-1.75)** | **1.64 (1.48-1.81)** | **0.61 (0.55-0.68)** | **2.74 (2.42-3.10)** | **CSW** | **3.68 (2.80-4.84)** |  |
| **0.43 (0.33-0.56)** | **0.44 (0.34-0.57)** | **0.17 (0.13-0.22)** | **0.74 (0.57-0.97)** | **0.27 (0.21-0.36)** | **BLOOD** |  |
|  | | | | | | |

Odds ratios and 95% confidence intervals for HIV-1 recombinants, relative to HIV-1 subtypes, are provided for each comparison between key populations. Key populations are provided in rows with the comparator key population indicated in each column, with odds ratios determined by dividing the key population in a row by the comparator in a column. For instance, all odds ratios in the left column use HET as a reference. Significant results are in bold.

Abbreviations: BLOOD = Blood/plasma transfusion associated infections, CSW = Commercial sex workers, HET = Heterosexual, MSM = Men who have sex with men, PWID = People who inject drugs, VERT = Vertical transmission (mother to child).

## Appendix 6: Regional associations of key populations and HIV-1 recombinants, 1990-2015.

### Latin America

| **HET** | **2.19 (1.49-3.21)** | **0.24 (0.17-0.33)** | **0.32 (0.26-0.40)** | **0.06 (0.04-0.09)** |
| --- | --- | --- | --- | --- |
| **0.46 (0.31-0.67)** | **MSM** | **0.11 (0.07-0.17)** | **0.15 (0.10-0.22)** | **0.03 (0.02-0.05)** |
| **4.16 (3.02-5.74)** | **9.11 (5.74-14.46)** | **PWID** | 1.34 (0.96-1.88) | **0.27 (0.17-0.42)** |
| **3.10 (2.50-3.85)** | **6.79 (4.56-10.10)** | 0.75 (0.53-1.05) | **VERT** | **0.20 (0.13-0.30)** |
| **15.58 (10.63-22.85)** | **34.10 (20.54-56.61)** | **3.74 (2.36-5.94)** | **5.02 (3.38-7.47)** | **CSW** |

### Western and central Europe, and North America (WCENA)

| **HET** | **5.55 (5.03-6.12)** | 1.07 (0.93-1.22) | **1.44 (1.14-1.81)** | **1.42 (1.04-1.95)** |
| --- | --- | --- | --- | --- |
| **0.18 (0.16-0.20)** | **MSM** | **0.19 (0.17-0.22)** | **0.26 (0.20-0.33)** | **0.26 (0.19-0.35)** |
| 0.94 (0.82-1.08) | **5.20 (4.48-6.03)** | **PWID** | **1.35 (1.04-1.74)** | 1.33 (0.95-1.86) |
| **0.69 (0.55-0.88)** | **3.85 (3.04-4.89)** | **0.74 (0.57-0.96)** | **VERT** | 0.99 (0.67-1.45) |
| **0.70 (0.51-0.96)** | **3.90 (2.83-5.37)** | 0.75 (0.54-1.05) | 1.01 (0.69-1.48) | **BLOOD** |

### Eastern Europe and central Asia (EECA)

| **HET** | **0.05 (0.02-0.16)** |
| --- | --- |
| **19.98 (6.30-63.34)** | **PWID** |

### South Asia (India)

| **HET** | 0.24 (0.06-1.02) |
| --- | --- |
| 4.18 (0.98-17.83) | **PWID** |

### Southeast Asia (SE Asia)

| **HET** | 1.50 (0.95-2.36) | **17.00 (14.02-20.61)** | **0.08 (0.01-0.59)** | 0.51 (0.16-1.62) |
| --- | --- | --- | --- | --- |
| 0.67 (0.42-1.05) | **MSM** | **11.32 (7.37-17.39)** | **0.05 (0.01-0.41)** | 0.34 (0.10-1.15) |
| **0.06 (0.05-0.07)** | **0.09 (0.06-0.14)** | **PWID** | **0.00 (0.00-0.03)** | **0.03 (0.01-0.09)** |
| **12.12 (1.69-86.98)** | **18.19 (2.44-135.45)** | **206.03 (28.88-1469.88)** | **VERT** | 6.19 (0.64-60.04) |
| 1.96 (0.62-6.21) | 2.94 (0.87-9.92) | **33.26 (10.58-104.59)** | 0.16 (0.02-1.56) | **CSW** |

###

### East Asia

| **HET** | **0.52 (0.43-0.62)** | **0.31 (0.24-0.40)** | 1.27 (0.49-3.29) | **25.47 (15.67-41.39)** |
| --- | --- | --- | --- | --- |
| **1.94 (1.60-2.34)** | **MSM** | **0.61 (0.50-0.74)** | 2.46 (0.96-6.30) | **49.28 (31.03-78.28)** |
| **3.20 (2.50-4.09)** | **1.65 (1.36-2.01)** | **PWID** | **4.06 (1.56-10.55)** | **81.43 (49.92-132.84)** |
| 0.79 (0.30-2.04) | 0.41 (0.16-1.04) | **0.25 (0.09-0.64)** | **VERT** | **20.06 (7.07-56.93)** |
| **0.04 (0.02-0.06)** | **0.02 (0.01-0.03)** | **0.01 (0.01-0.02)** | **0.05 (0.02-0.14)** | **BLOOD** |

### West Africa

| **HET** | **0.69 (0.55-0.88)** | **0.30 (0.18-0.49)** | **1.48 (1.23-1.78)** |
| --- | --- | --- | --- |
| **1.44 (1.14-1.82)** | **MSM** | **0.42 (0.24-0.74)** | **2.13 (1.58-2.85)** |
| **3.39 (2.04-5.63)** | **2.35 (1.35-4.10)** | **VERT** | **5.00 (2.92-8.56)** |
| **0.68 (0.56-0.82)** | **0.47 (0.35-0.63)** | **0.20 (0.12-0.34)** | **CSW** |

### East Africa

| **HET** | 0.93 (0.73-1.18) | **0.30 (0.25-0.35)** |
| --- | --- | --- |
| 1.08 (0.85-1.38) | **VERT** | **0.32 (0.24-0.42)** |
| **3.36 (2.85-3.97)** | **3.11 (2.35-4.11)** | **CSW** |

### Central Africa

| **HET** | 1.25 (0.91-1.72) |
| --- | --- |
| 0.80 (0.58-1.10) | **VERT** |

### Southern Africa

| **HET** | 0.89 (0.28-2.83) | **7.25 (1.78-29.54)** |
| --- | --- | --- |
| 1.13 (0.35-3.60) | **MSM** | **8.18 (1.36-49.20)** |
| **0.14 (0.03-0.56)** | **0.12 (0.02-0.74)** | **VERT** |

For each region, odds ratios and 95% confidence intervals are provided for each comparison. Key populations are provided in rows with the comparator key population indicated in each column. Odds ratios can be determined by dividing the key population in a row by the comparator in a column. For instance, all odds ratios in the left column use HET as a reference. Significant results are in bold.

Abbreviations: BLOOD = Blood/plasma transfusion associated infections, CSW = Commercial sex workers, HET = Heterosexual, MSM = Men who have sex with men, PWID = People who inject drugs, VERT = Vertical transmission (mother to child).

##
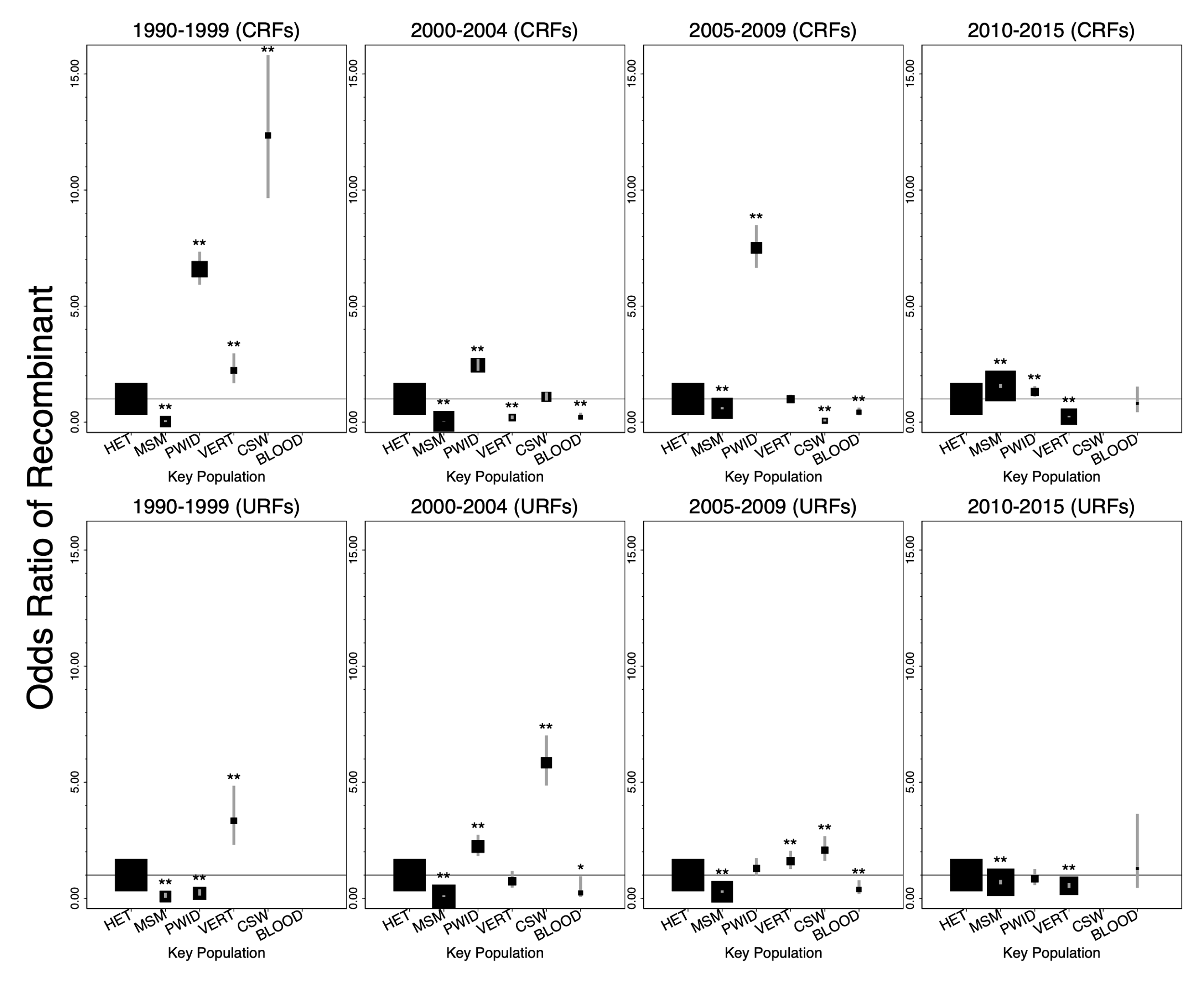
Appendix 7: Global associations of HIV-1 CRFs and URFs for key populations, relative to heterosexual people, for each time period.

Odds ratios for HIV-1 CRFs and URFs, compared to HIV-1 subtypes, of key populations relative to heterosexual people for each time period (1990-1999, 2000-2004, 2005-2009, 2010-2015).

Error bars represent the 95% confidence intervals. Square areas are proportional to the number of participants in each key population analysed. (* P<0.05, ** P<0.01)

Abbreviations: BLOOD = Blood/plasma transfusion associated infections, CRF = Circulating Recombinant Form, CSW = Commercial sex workers, HET = Heterosexual, MSM = Men who have sex with men, PWID = People who inject drugs, URF = Unique Recombinant Form, VERT = Vertical transmission (mother to child).

## Appendix 8: Global associations of HIV-1 recombinants, CRFs, and URFs for key populations, relative to heterosexual people, for each time period.

|  | **HET** | **MSM** | **PWID** | **VERT** | **CSW** | **BLOOD** |
| --- | --- | --- | --- | --- | --- | --- |
| **Recombinants** | | | | | | |
| GLOBAL (1990-2015) | 1 | 0.97 (0.93-1.01) | **2.60 (2.46-2.74)** | **0.58 (0.54-0.63)** | **1.59 (1.44-1.75)** | **0.43 (0.33-0.56)** |
| 1990-1999 | 1 | **0.04 (0.02-0.09)** | **4.88 (4.41-5.41)** | **2.53 (1.99-3.22)** | **9.04 (7.08-11.54)** | - |
| 2000-2004 | 1 | **0.05 (0.04-0.06)** | **2.42 (2.19-2.68)** | **0.28 (0.21-0.38)** | **1.82 (1.60-2.09)** | **0.22 (0.12-0.39)** |
| 2005-2009 | 1 | **0.53 (0.49-0.57)** | **6.13 (5.44-6.92)** | 1.13 (0.97-1.31) | **0.51 (0.40-0.64)** | **0.42 (0.30-0.59)** |
| 2010-2015 | 1 | **1.43 (1.35-1.51)** | **1.23 (1.04-1.46)** | **0.28 (0.25-0.32)** | - | 0.88 (0.49-1.59) |
| **CRFs** | | | | | | |
| GLOBAL (1990-2015) | 1 | **1.09 (1.05-1.14)** | **2.99 (2.83-3.16)** | **0.49 (0.44-0.53)** | 1.11 (0.98-1.25) | **0.43 (0.32-0.56**) |
| 1990-1999 | 1 | **0.03 (0.01-0.09)** | **6.59 (5.92-7.34)** | **2.23 (1.68-2.97)** | **2.23 (1.68-2.97)** | - |
| 2000-2004 | 1 | **0.04 (0.03-0.05)** | **2.46 (2.21-2.73)** | **0.20 (0.13-0.29)** | 1.09 (0.93-1.29) | **0.21 (0.11-0.41)** |
| 2005-2009 | 1 | **0.60 (0.55-0.65)** | **7.51 (6.64-8.49)** | 0.99 (0.83-1.18) | **0.06 (0.03-0.12)** | **0.43 (0.30-0.63)** |
| 2010-2015 | 1 | **1.56 (1.47-1.65)** | **1.30 (1.09-1.55)** | **0.24 (0.21-0.27)** | - | 0.81 (0.43-1.53) |
| **URFs** | | | | | | |
| GLOBAL (1990-2015) | 1 | **0.44 (0.40-0.48)** | 0.95 (0.82-1.09) | 0.97 (0.85-1.11) | **3.61 (3.15-4.13)** | **0.45 (0.26-0.77)** |
| 1990-1999 | 1 | **0.08 (0.02-0.24)** | **0.21 (0.12-0.39)** | **3.34 (2.30-4.85)** | - | - |
| 2000-2004 | 1 | **0.08 (0.05-0.13)** | **2.23 (1.82-2.74)** | 0.73 (0.46-1.18) | **5.84 (4.85-7.01)** | **0.23 (0.06-0.95)** |
| 2005-2009 | 1 | **0.28 (0.23-0.34)** | 1.29 (0.96-1.73) | **1.60 (1.26-2.04)** | **2.07 (1.61-2.67)** | **0.38 (0.19-0.78)** |
| 2010-2015 | 1 | **0.69 (0.60-0.79)** | 0.84 (0.56-1.26) | **0.54 (0.44-0.66)** | - | 1.28 (0.45-3.64) |

Odds ratios for HIV-1 recombinants, CRFs, and URFs, compared to HIV-1 subtypes, of key populations relative to heterosexual people in 1990-2015 and in each time period (1990-1999, 2000-2004, 2005-2009, 2010-2015). Significant results are bolded.

Abbreviations: BLOOD = Blood/plasma transfusion associated infections, CRF = Circulating Recombinant Form, CSW = Commercial sex workers, HET = Heterosexual, MSM = Men who have sex with men, PWID = People who inject drugs, URF = Unique Recombinant Form, VERT = Vertical transmission (mother to child).

## Appendix 9: Global distribution of HIV-1 subtypes, individual CRFs, and URFs among key populations, 1990-2015.

|  | **A** | **B** | **C** | **D** | **F** | **G** | **H** | **J** | **K** | **CRF01_AE** |
| --- | --- | --- | --- | --- | --- | --- | --- | --- | --- | --- |
| **HET** | 8,340 (18.6%) | 5,889 (13.1%) | 11,408 (25.4%) | 3,222 (7.2%) | 580 (1.3%) | 1,729 (3.8%) | 182 (0.4%) | 64 (0.1%) | 22 (0.0%) | 4,140 (9.2%) |
| **MSM** | 157 (0.8%) | 12,937 (65.2%) | 561 (2.8%) | 12 (0.1%) | 219 (1.1%) | 104 (0.5%) | 4 (0.0%) | 1 (0.0%) | 7 (0.0%) | 3,154 (15.9%) |
| **PWID** | 794 (12.7%) | 1,846 (29.6%) | 169 (2.7%) | 8 (0.1%) | 116 (1.9%) | 13 (0.2%) | 0 (0.0%) | 0 (0.0%) | 0 (0.0%) | 1,363 (21.9%) |
| **VERT** | 578 (14.0%) | 824 (20.0%) | 1,627 (39.4%) | 152 (3.7%) | 71 (1.7%) | 45 (1.1%) | 5 (0.1%) | 3 (0.1%) | 0 (0.0%) | 328 (7.9%) |
| **CSW** | 439 (26.1%) | 47 (2.8%) | 367 (21.8%) | 72 (4.3%) | 19 (1.1%) | 56 (3.3%) | 0 (0.0%) | 2 (0.1%) | 0 (0.0%) | 155 (9.2%) |
| **BLOOD** | 11 (2.5%) | 270 (60.3%) | 19 (4.2%) | 5 (1.1%) | 70 (15.6%) | 3 (0.7%) | 0 (0.0%) | 0 (0.0%) | 0 (0.0%) | 18 (4.0%) |
|  | **CRF02_AG** | **CRF03_AB** | **CRF04_cpx** | **CRF05_DF** | **CRF06_cpx** | **CRF07_BC** | **CRF08_BC** | **CRF09_cpx** | **CRF10_CD** | **CRF11_cpx** |
| **HET** | 4,995 (11.1%) | 21 (0.0%) | 3 (0.0%) | 34 (0.1%) | 600 (1.3%) | 167 (0.4%) | 93 (0.2%) | 51 (0.1%) | 76 (0.2%) | 263 (0.6%) |
| **MSM** | 446 (2.2%) | 4 (0.0%) | 0 (0.0%) | 8 (0.0%) | 16 (0.1%) | 1,320 (6.7%) | 5 (0.0%) | 3 (0.0%) | 0 (0.0%) | 0 (0.0%) |
| **PWID** | 8 (0.1%) | 65 (1.0%) | 0 (0.0%) | 0 (0.0%) | 153 (2.5%) | 989 (15.9%) | 226 (3.6%) | 0 (0.0%) | 0 (0.0%) | 31 (0.5%) |
| **VERT** | 136 (3.3%) | 0 (0.0%) | 0 (0.0%) | 1 (0.0%) | 22 (0.5%) | 6 (0.1%) | 5 (0.1%) | 2 (0.0%) | 4 (0.1%) | 0 (0.0%) |
| **CSW** | 196 (11.6%) | 0 (0.0%) | 0 (0.0%) | 0 (0.0%) | 28 (1.7%) | 0 (0.0%) | 0 (0.0%) | 8 (0.5%) | 0 (0.0%) | 0 (0.0%) |
| **BLOOD** | 25 (5.6%) | 0 (0.0%) | 0 (0.0%) | 0 (0.0%) | 3 (0.7%) | 7 (1.6%) | 3 (0.7%) | 0 (0.0%) | 0 (0.0%) | 0 (0.0%) |
|  | **CRF12_BF** | **CRF13_cpx** | **CRF14_BG** | **CRF15_01B** | **CRF16_A2D** | **CRF17_BF** | **CRF18_cpx** | **CRF19_cpx** | **CRF20_BG** | **CRF21_A2D** |
| **HET** | 24 (0.1%) | 62 (0.1%) | 2 (0.0%) | 28 (0.1%) | 7 (0.0%) | 0 (0.0%) | 35 (0.1%) | 7 (0.0%) | 2 (0.0%) | 1 (0.0%) |
| **MSM** | 42 (0.2%) | 0 (0.0%) | 7 (0.0%) | 1 (0.0%) | 0 (0.0%) | 10 (0.1%) | 44 (0.2%) | 100 (0.5%) | 1 (0.0%) | 0 (0.0%) |
| **PWID** | 12 (0.2%) | 0 (0.0%) | 28 (0.4%) | 0 (0.0%) | 0 (0.0%) | 0 (0.0%) | 0 (0.0%) | 0 (0.0%) | 0 (0.0%) | 0 (0.0%) |
| **VERT** | 17 (0.4%) | 1 (0.0%) | 0 (0.0%) | 0 (0.0%) | 2 (0.0%) | 0 (0.0%) | 3 (0.1%) | 0 (0.0%) | 0 (0.0%) | 0 (0.0%) |
| **CSW** | 0 (0.0%) | 0 (0.0%) | 0 (0.0%) | 0 (0.0%) | 0 (0.0%) | 0 (0.0%) | 0 (0.0%) | 0 (0.0%) | 0 (0.0%) | 0 (0.0%) |
| **BLOOD** | 0 (0.0%) | 0 (0.0%) | 0 (0.0%) | 0 (0.0%) | 0 (0.0%) | 0 (0.0%) | 0 (0.0%) | 0 (0.0%) | 0 (0.0%) | 0 (0.0%) |
|  | **CRF22_01A1** | **CRF23_BG** | **CRF24_BG** | **CRF25_cpx** | **CRF26_AU** | **CRF27_cpx** | **CRF28_BF** | **CRF29_BF** | **CRF31_BC** | **CRF32_06A1** |
| **HET** | 122 (0.3%) | 0 (0.0%) | 3 (0.0%) | 29 (0.1%) | 3 (0.0%) | 2 (0.0%) | 3 (0.0%) | 1 (0.0%) | 49 (0.1%) | 2 (0.0%) |
| **MSM** | 0 (0.0%) | 88 (0.4%) | 1 (0.0%) | 0 (0.0%) | 0 (0.0%) | 0 (0.0%) | 3 (0.0%) | 0 (0.0%) | 14 (0.1%) | 0 (0.0%) |
| **PWID** | 0 (0.0%) | 0 (0.0%) | 0 (0.0%) | 0 (0.0%) | 0 (0.0%) | 0 (0.0%) | 0 (0.0%) | 0 (0.0%) | 0 (0.0%) | 1 (0.0%) |
| **VERT** | 0 (0.0%) | 0 (0.0%) | 6 (0.1%) | 0 (0.0%) | 5 (0.1%) | 0 (0.0%) | 7 (0.2%) | 0 (0.0%) | 2 (0.0%) | 0 (0.0%) |
| **CSW** | 0 (0.0%) | 0 (0.0%) | 0 (0.0%) | 0 (0.0%) | 0 (0.0%) | 0 (0.0%) | 0 (0.0%) | 0 (0.0%) | 0 (0.0%) | 0 (0.0%) |
| **BLOOD** | 0 (0.0%) | 0 (0.0%) | 0 (0.0%) | 0 (0.0%) | 0 (0.0%) | 0 (0.0%) | 0 (0.0%) | 0 (0.0%) | 0 (0.0%) | 0 (0.0%) |
|  | **CRF33_01B** | **CRF34_01B** | **CRF35_AD** | **CRF36_cpx** | **CRF37_cpx** | **CRF39_BF** | **CRF40_BF** | **CRF43_02G** | **CRF44_BF** | **CRF45_AKU** |
| **HET** | 11 (0.0%) | 2 (0.0%) | 15 (0.0%) | 16 (0.0%) | 42 (0.1%) | 2 (0.0%) | 0 (0.0%) | 4 (0.0%) | 2 (0.0%) | 20 (0.0%) |
| **MSM** | 0 (0.0%) | 0 (0.0%) | 0 (0.0%) | 0 (0.0%) | 0 (0.0%) | 0 (0.0%) | 0 (0.0%) | 0 (0.0%) | 0 (0.0%) | 0 (0.0%) |
| **PWID** | 91 (1.5%) | 0 (0.0%) | 40 (0.6%) | 0 (0.0%) | 0 (0.0%) | 0 (0.0%) | 0 (0.0%) | 0 (0.0%) | 0 (0.0%) | 0 (0.0%) |
| **VERT** | 0 (0.0%) | 0 (0.0%) | 0 (0.0%) | 0 (0.0%) | 0 (0.0%) | 2 (0.0%) | 3 (0.1%) | 0 (0.0%) | 0 (0.0%) | 8 (0.2%) |
| **CSW** | 0 (0.0%) | 0 (0.0%) | 0 (0.0%) | 0 (0.0%) | 0 (0.0%) | 0 (0.0%) | 0 (0.0%) | 0 (0.0%) | 0 (0.0%) | 0 (0.0%) |
| **BLOOD** | 0 (0.0%) | 0 (0.0%) | 0 (0.0%) | 0 (0.0%) | 0 (0.0%) | 0 (0.0%) | 0 (0.0%) | 0 (0.0%) | 0 (0.0%) | 0 (0.0%) |
|  | **CRF49_cpx** | **CRF50_A1D** | **CRF55_01B** | **CRF59_01B** | **CRF63_02A1** | **CRF67_01B** | **CRF68_01B** | **Total URFs** | **Total** |  |
| **HET** | 3 (0.0%) | 0 (0.0%) | 0 (0.0%) | 0 (0.0%) | 0 (0.0%) | 0 (0.0%) | 0 (0.0%) | 2,574 (5.7%) | 44,952 (100%) |  |
| **MSM** | 0 (0.0%) | 2 (0.0%) | 43 (0.2%) | 1 (0.0%) | 0 (0.0%) | 12 (0.1%) | 5 (0.0%) | 503 (2.5%) | 19,835 (100%) |  |
| **PWID** | 0 (0.0%) | 0 (0.0%) | 0 (0.0%) | 0 (0.0%) | 55 (0.9%) | 0 (0.0%) | 0 (0.0%) | 228 (3.7%) | 6,236 (100%) |  |
| **VERT** | 0 (0.0%) | 0 (0.0%) | 0 (0.0%) | 0 (0.0%) | 0 (0.0%) | 0 (0.0%) | 0 (0.0%) | 263 (6.4%) | 4,128 (100%) |  |
| **CSW** | 0 (0.0%) | 0 (0.0%) | 0 (0.0%) | 0 (0.0%) | 0 (0.0%) | 0 (0.0%) | 0 (0.0%) | 296 (17.6%) | 1,685 (100%) |  |
| **BLOOD** | 0 (0.0%) | 0 (0.0%) | 0 (0.0%) | 0 (0.0%) | 0 (0.0%) | 0 (0.0%) | 0 (0.0%) | 14 (3.1%) | 448 (100%) |  |

Global distribution of HIV-1 subtypes, individual CRFs, and URFs within key populations in 1990-2015.

Abbreviations: BLOOD = Blood/plasma transfusion associated infections, CRF = Circulating Recombinant Form, CSW = Commercial sex workers, HET = Heterosexual, MSM = Men who have sex with men, PWID = People who inject drugs, URF = Unique Recombinant Form, VERT = Vertical transmission (mother to child).

## Appendix 10: Regional distribution of HIV-1 subtypes, CRFs, and URFs among key populations, 1990-2015.

|  | **HIV-1 Subtypes** | | | | | | | | |  | **CRFs** | | | **URFs** | **Total CRFs*** | **Total Recombinants†** | **Total‡** | |
| --- | --- | --- | --- | --- | --- | --- | --- | --- | --- | --- | --- | --- | --- | --- | --- | --- | --- | --- |
|  | **A** | **B** | **C** | **D** | **F** | **G** | **H** | **J** | **K** |  | **CRF01_AE** | **CRF02_AG** | **Other** |  |  |  |  | |
| **GLOBAL (1990-2015)** | | | | | | | | | | | | | | | | | | |
| HET | 8,340 (18.6%) | 5,889 (13.1%) | 11,408 (25.4%) | 3,222 (7.2%) | 580 (1.3%) | 1,729 (3.8%) | 182 (0.4%) | 64 (0.1%) | 22 (0.0%) |  | 4,140 (9.2%) | 4,995 (11.1%) | 1,807 (4%) | 2,574 (5.7%) | 10,942 (24.3%) | 13,516 (30.1%) | 44,952 (100%) | |
| MSM | 157 (0.8%) | 12,937 (65.2%) | 561 (2.8%) | 12 (0.1%) | 219 (1.1%) | 104 (0.5%) | 4 (0.0%) | 1 (0.0%) | 7 (0.0%) |  | 3,154 (15.9%) | 446 (2.2%) | 1,730 (8.7%) | 503 (2.5%) | 5,330 (26.9%) | 5,833 (29.4%) | 19,835 (100%) | |
| PWID | 794 (12.7%) | 1,846 (29.6%) | 169 (2.7%) | 8 (0.1%) | 116 (1.9%) | 13 (0.2%) | 0 (0.0%) | 0 (0.0%) | 0 (0.0%) |  | 1,363 (21.9%) | 8 (0.1%) | 1,691 (27.1%) | 228 (3.7%) | 3,062 (49.1%) | 3,290 (52.8%) | 6,236 (100%) | |
| VERT | 578 (14%) | 824 (20%) | 1,627 (39.4%) | 152 (3.7%) | 71 (1.7%) | 45 (1.1%) | 5 (0.1%) | 3 (0.1%) | 0 (0.0%) |  | 328 (7.9%) | 136 (3.3%) | 96 (2.3%) | 263 (6.4%) | 560 (13.6%) | 823 (19.9%) | 4,128 (100%) | |
| CSW | 439 (26.1%) | 47 (2.8%) | 367 (21.8%) | 72 (4.3%) | 19 (1.1%) | 56 (3.3%) | 2 (0.1%) | 0 (0.0%) | 0 (0.0%) |  | 155 (9.2%) | 196 (11.6%) | 36 (2.1%) | 296 (17.6%) | 387 (23%) | 683 (40.5%) | 1,685 (100%) | |
| BLOOD | 11 (2.5%) | 270 (60.3%) | 19 (4.2%) | 5 (1.1%) | 70 (15.6%) | 3 (0.7%) | 0 (0.0%) | 0 (0.0%) | 0 (0.0%) |  | 18 (4%) | 25 (5.6%) | 13 (2.9%) | 14 (3.1%) | 56 (12.5%) | 70 (15.6%) | 448 (100%) | |
| **Caribbean** | | | | | | | | | | | | | | | | | | |
| HET | 0 (0.0%) | 662 (100%) | 0 (0.0%) | 0 (0.0%) | 0 (0.0%) | 0 (0.0%) | 0 (0.0%) | 0 (0.0%) | 0 (0.0%) |  | 0 (0.0%) | 0 (0.0%) | 0 (0.0%) | 0 (0.0%) | 0 (0.0%) | 0 (0.0%) | 662 (100%) | |
| MSM | 1 (0.2%) | 145 (34.4%) | 11 (2.6%) | 0 (0.0%) | 1 (0.2%) | 11 (2.6%) | 2 (0.5%) | 1 (0.2%) | 0 (0.0%) |  | 0 (0.0%) | 0 (0.0%) | 234 (55.6%) | 15 (3.6%) | 234 (55.6%) | 249 (59.1%) | 421 (100%) | |
| **Latin America** | | | | | | | | | | | | | | | | | | |
| HET | 0 (0.0%) | 2,486 (79.0%) | 207 (6.6%) | 6 (0.2%) | 215 (6.8%) | 0 (0.0%) | 0 (0.0%) | 0 (0.0%) | 0 (0.0%) |  | 0 (0.0%) | 4 (0.1%) | 67 (2.1%) | 161 (5.1%) | 71 (2.3%) | 232 (7.4%) | 3,146 (100%) | |
| MSM | 0 (0.0%) | 796 (90.1%) | 28 (3.2%) | 0 (0.0%) | 28 (3.2%) | 0 (0.0%) | 0 (0.0%) | 0 (0.0%) | 0 (0.0%) |  | 0 (0.0%) | 0 (0.0%) | 0 (0.0%) | 31 (3.5%) | 0 (0.0%) | 31 (3.5%) | 883 (100%) | |
| PWID | 0 (0.0%) | 131 (54.4%) | 27 (11.2%) | 1 (0.4%) | 22 (9.1%) | 0 (0.0%) | 0 (0.0%) | 0 (0.0%) | 0 (0.0%) |  | 0 (0.0%) | 0 (0.0%) | 12 (5.0%) | 48 (19.9%) | 12 (5%) | 60 (24.9%) | 241 (100%) | |
| VERT | 1 (0.1%) | 556 (67.1%) | 46 (5.6%) | 0 (0.0%) | 61 (7.4%) | 0 (0.0%) | 0 (0.0%) | 0 (0.0%) | 0 (0.0%) |  | 0 (0.0%) | 0 (0.0%) | 30 (3.6%) | 134 (16.2%) | 30 (3.6%) | 164 (19.8%) | 828 (100%) | |
| CSW | 0 (0.0%) | 34 (28.1%) | 4 (3.3%) | 0 (0.0%) | 16 (13.2%) | 0 (0.0%) | 0 (0.0%) | 0 (0.0%) | 0 (0.0%) |  | 0 (0.0%) | 0 (0.0%) | 0 (0.0%) | 67 (55.4%) | 0 (0.0%) | 67 (55.4%) | 121 (100%) | |
| **Western and central Europe, and North America** | | | | | | | | | | | | | | | | | | |
| HET | 622 (9.6%) | 2,249 (34.7%) | 1,518 (23.4%) | 174 (2.7%) | 83 (1.3%) | 247 (3.8%) | 10 (0.2%) | 17 (0.3%) | 6 (0.1%) |  | 295 (4.5%) | 899 (13.9%) | 89 (1.4%) | 276 (4.3%) | 1,283 (19.8%) | 1,559 (24%) | 6,485 (100%) | |
| MSM | 143 (1.2%) | 10,451 (89.1%) | 243 (2.1%) | 12 (0.1%) | 186 (1.6%) | 49 (0.4%) | 2 (0.0%) | 0 (0.0%) | 7 (0.1%) |  | 144 (1.2%) | 274 (2.3%) | 101 (0.9%) | 114 (1.0%) | 519 (4.4%) | 633 (5.4%) | 11,726 (100%) | |
| PWID | 165 (11.9%) | 755 (54.3%) | 46 (3.3%) | 0 (0.0%) | 94 (6.8%) | 12 (0.9%) | 0 (0.0%) | 0 (0.0%) | 0 (0.0%) |  | 63 (4.5%) | 8 (0.6%) | 216 (15.5%) | 31 (2.2%) | 287 (20.6%) | 318 (22.9%) | 1,390 (100%) | |
| VERT | 49 (9.5%) | 257 (49.8%) | 87 (16.9%) | 8 (1.6%) | 6 (1.2%) | 15 (2.9%) | 1 (0.2%) | 0 (0.0%) | 0 (0.0%) |  | 5 (1.0%) | 65 (12.6%) | 11 (2.1%) | 12 (2.3%) | 81 (15.7%) | 93 (18%) | 516 (100%) | |
| BLOOD | 11 (4.1%) | 112 (41.6%) | 19 (7.1%) | 5 (1.9%) | 70 (26.0%) | 3 (1.1%) | 0 (0.0%) | 0 (0.0%) | 0 (0.0%) |  | 9 (3.3%) | 25 (9.3%) | 3 (1.1%) | 12 (4.5%) | 37 (13.8%) | 49 (18.2%) | 269 (100%) | |
|  | | | | | | | | | | | | | | | | | | |
| **Eastern Europe and central Asia** | | | | | | | | | | | | | | | | | | |
| HET | 193 (73.4%) | 34 (12.9%) | 15 (5.7%) | 10 (3.8%) | 0 (0.0%) | 6 (2.3%) | 2 (0.8%) | 0 (0.0%) | 0 (0.0%) |  | 1 (0.4%) | 0 (0.0%) | 0 (0.0%) | 2 (0.8%) | 1 (0.4%) | 3 (1.1%) | 263 (100%) | |
| MSM | 3 (4.0%) | 72 (96.0%) | 0 (0.0%) | 0 (0.0%) | 0 (0.0%) | 0 (0.0%) | 0 (0.0%) | 0 (0.0%) | 0 (0.0%) |  | 0 (0.0%) | 0 (0.0%) | 0 (0.0%) | 0 (0.0%) | 0 (0.0%) | 0 (0.0%) | 75 (100%) | |
| PWID | 508 (73.2%) | 56 (8.1%) | 0 (0.0%) | 0 (0.0%) | 0 (0.0%) | 0 (0.0%) | 0 (0.0%) | 0 (0.0%) | 0 (0.0%) |  | 0 (0.0%) | 0 (0.0%) | 119 (17.1%) | 11 (1.6%) | 119 (17.1%) | 130 (18.7%) | 694 (100%) | |
| VERT | 4 (50.0%) | 0 (0.0%) | 2 (25.0%) | 0 (0.0%) | 0 (0.0%) | 2 (25.0%) | 0 (0.0%) | 0 (0.0%) | 0 (0.0%) |  | 0 (0.0%) | 0 (0.0%) | 0 (0.0%) | 0 (0.0%) | 0 (0.0%) | 0 (0.0%) | 8 (100%) | |
| **South Asia** | | | | | | | | | | | | | | | | | |  |
| HET | 5 (0.9%) | 12 (2.2%) | 519 (95.9%) | 0 (0.0%) | 0 (0.0%) | 0 (0.0%) | 0 (0.0%) | 0 (0.0%) | 0 (0.0%) |  | 1 (0.2%) | 0 (0.0%) | 0 (0.0%) | 4 (0.7%) | 1 (0.2%) | 5 (0.9%) | 541 (100%) |  |
| PWID | 0 (0.0%) | 5 (6.2%) | 72 (90.0%) | 0 (0.0%) | 0 (0.0%) | 0 (0.0%) | 0 (0.0%) | 0 (0.0%) | 0 (0.0%) |  | 0 (0.0%) | 0 (0.0%) | 0 (0.0%) | 3 (3.8%) | 0 (0.0%) | 3 (3.8%) | 80 (100%) |  |
| CSW | 0 (0.0%) | 0 (0.0%) | 114 (100%) | 0 (0.0%) | 0 (0.0%) | 0 (0.0%) | 0 (0.0%) | 0 (0.0%) | 0 (0.0%) |  | 0 (0.0%) | 0 (0.0%) | 0 (0.0%) | 0 (0.0%) | 0 (0.0%) | 0 (0.0%) | 114 (100%) |  |
| **Southeast Asia** | | | | | | | | | | | | | | | | | |  |
| HET | 0 (0.0%) | 132 (3.6%) | 1 (0.0%) | 1 (0.0%) | 0 (0.0%) | 0 (0.0%) | 0 (0.0%) | 0 (0.0%) | 0 (0.0%) |  | 3,433 (94.1%) | 1 (0.0%) | 37 (1.0%) | 44 (1.2%) | 3,471 (95.1%) | 3,515 (96.3%) | 3,649 (100%) |  |
| MSM | 0 (0.0%) | 23 (5.4%) | 0 (0.0%) | 0 (0.0%) | 0 (0.0%) | 0 (0.0%) | 0 (0.0%) | 0 (0.0%) | 0 (0.0%) |  | 349 (82.1%) | 0 (0.0%) | 0 (0.0%) | 53 (12.5%) | 349 (82.1%) | 402 (94.6%) | 425 (100%) |  |
| PWID | 69 (3.1%) | 788 (35.9%) | 3 (0.1%) | 2 (0.1%) | 0 (0.0%) | 1 (0.0%) | 0 (0.0%) | 0 (0.0%) | 0 (0.0%) |  | 1,138 (51.8%) | 0 (0.0%) | 91 (4.1%) | 103 (4.7%) | 1,229 (56%) | 1,332 (60.7%) | 2,195 (100%) |  |
| VERT | 0 (0.0%) | 1 (0.3%) | 0 (0.0%) | 0 (0.0%) | 0 (0.0%) | 0 (0.0%) | 0 (0.0%) | 0 (0.0%) | 0 (0.0%) |  | 318 (99.7%) | 0 (0.0%) | 0 (0.0%) | 0 (0.0%) | 318 (99.7%) | 318 (99.7%) | 319 (100%) |  |
| CSW | 0 (0.0%) | 3 (1.9%) | 0 (0.0%) | 0 (0.0%) | 0 (0.0%) | 0 (0.0%) | 0 (0.0%) | 0 (0.0%) | 0 (0.0%) |  | 154 (98.1%) | 0 (0.0%) | 0 (0.0%) | 0 (0.0%) | 154 (98.1%) | 154 (98.1%) | 157 (100%) |  |
| **East Asia** | | | | | | | | | | | | | | | | | | |
| HET | 3 (0.4%) | 151 (19.8%) | 18 (2.4%) | 0 (0.0%) | 0 (0.0%) | 2 (0.3%) | 0 (0.0%) | 0 (0.0%) | 0 (0.0%) |  | 303 (39.7%) | 6 (0.8%) | 255 (33.4%) | 25 (3.3%) | 564 (73.9%) | 589 (77.2%) | 763 (100%) | |
| MSM | 0 (0.0%) | 643 (13.2%) | 2 (0.0%) | 0 (0.0%) | 0 (0.0%) | 2 (0.0%) | 0 (0.0%) | 0 (0.0%) | 0 (0.0%) |  | 2,629 (53.8%) | 4 (0.1%) | 1,392 (28.5%) | 213 (4.4%) | 4,025 (82.4%) | 4,238 (86.8%) | 4,885 (100%) | |
| PWID | 0 (0.0%) | 111 (7.2%) | 19 (1.2%) | 0 (0.0%) | 0 (0.0%) | 0 (0.0%) | 0 (0.0%) | 0 (0.0%) | 0 (0.0%) |  | 162 (10.5%) | 0 (0.0%) | 1,213 (78.9%) | 32 (2.1%) | 1,375 (89.5%) | 1,407 (91.5%) | 1,537 (100%) | |
| VERT | 0 (0.0%) | 6 (27.3%) | 0 (0.0%) | 0 (0.0%) | 0 (0.0%) | 0 (0.0%) | 0 (0.0%) | 0 (0.0%) | 0 (0.0%) |  | 5 (22.7%) | 0 (0.0%) | 10 (45.5%) | 1 (4.5%) | 15 (68.2%) | 16 (72.7%) | 22 (100%) | |
| BLOOD | 0 (0.0%) | 158 (88.3%) | 0 (0.0%) | 0 (0.0%) | 0 (0.0%) | 0 (0.0%) | 0 (0.0%) | 0 (0.0%) | 0 (0.0%) |  | 9 (5.0%) | 0 (0.0%) | 10 (5.6%) | 2 (1.1%) | 19 (10.6%) | 21 (11.7%) | 179 (100%) | |
| **Oceania** | | | | | | | | | | | | | | | | | |  |
| HET | 1 (1.1%) | 21 (22.6%) | 70 (75.3%) | 0 (0.0%) | 0 (0.0%) | 1 (1.1%) | 0 (0.0%) | 0 (0.0%) | 0 (0.0%) |  | 0 (0.0%) | 0 (0.0%) | 0 (0.0%) | 0 (0.0%) | 0 (0.0%) | 0 (0.0%) | 93 (100%) |  |
| MSM | 5 (0.6%) | 761 (92.1%) | 10 (1.2%) | 0 (0.0%) | 0 (0.0%) | 1 (0.1%) | 0 (0.0%) | 0 (0.0%) | 0 (0.0%) |  | 32 (3.9%) | 5 (0.6%) | 0 (0.0%) | 12 (1.5%) | 37 (4.5%) | 49 (5.9%) | 826 (100%) |  |
| **Middle East and North Africa** | | | | | | | | | | | | | | | | | |  |
| HET | 0 (0.0%) | 46 (64.8%) | 0 (0.0%) | 4 (5.6%) | 0 (0.0%) | 0 (0.0%) | 0 (0.0%) | 0 (0.0%) | 0 (0.0%) |  | 0 (0.0%) | 18 (25.4%) | 3 (4.2%) | 0 (0.0%) | 21 (29.6%) | 21 (29.6%) | 71 (100%) |  |
| PWID | 0 (0.0%) | 0 (0.0%) | 0 (0.0%) | 0 (0.0%) | 0 (0.0%) | 0 (0.0%) | 0 (0.0%) | 0 (0.0%) | 0 (0.0%) |  | 0 (0.0%) | 0 (0.0%) | 40 (100.0%) | 0 (0.0%) | 40 (100%) | 40 (100%) | 40 (100%) |  |
| **West Africa** | | | | | | | | | | | | | | | | | |  |
| HET | 1,951 (21.0%) | 48 (0.5%) | 87 (0.9%) | 194 (2.1%) | 170 (1.8%) | 1,228 (13.2%) | 15 (0.2%) | 4 (0.0%) | 5 (0.1%) |  | 43 (0.5%) | 3,849 (41.4%) | 950 (10.2%) | 742 (8.0%) | 4,842 (52.1%) | 5,584 (60.1%) | 9,286 (100%) |  |
| MSM | 1 (0.3%) | 13 (3.9%) | 50 (15.0%) | 0 (0.0%) | 0 (0.0%) | 41 (12.3%) | 0 (0.0%) | 0 (0.0%) | 0 (0.0%) |  | 0 (0.0%) | 163 (48.9%) | 3 (0.9%) | 62 (18.6%) | 166 (49.8%) | 228 (68.5%) | 333 (100%) |  |
| VERT | 6 (5.5%) | 0 (0.0%) | 0 (0.0%) | 0 (0.0%) | 0 (0.0%) | 12 (10.9%) | 0 (0.0%) | 0 (0.0%) | 0 (0.0%) |  | 0 (0.0%) | 63 (57.3%) | 17 (15.5%) | 12 (10.9%) | 80 (72.7%) | 92 (83.6%) | 110 (100%) |  |
| CSW | 131 (28.2%) | 10 (2.2%) | 15 (3.2%) | 13 (2.8%) | 3 (0.6%) | 56 (12.0%) | 2 (0.4%) | 0 (0.0%) | 0 (0.0%) |  | 1 (0.2%) | 196 (42.2%) | 36 (7.7%) | 2 (0.4%) | 233 (50.1%) | 235 (50.5%) | 465 (100%) |  |
| **East Africa** | | | | | | | | | | | | | | | | | | |
| HET | 4,568 (48.9%) | 5 (0.1%) | 1,224 (13.1%) | 2,540 (27.2%) | 8 (0.1%) | 52 (0.6%) | 3 (0.0%) | 2 (0.0%) | 1 (0.0%) |  | 21 (0.2%) | 4 (0.0%) | 86 (0.9%) | 833 (8.9%) | 111 (1.2%) | 944 (10.1%) | 9,347 (100%) | |
| PWID | 52 (88.1%) | 0 (0.0%) | 2 (3.4%) | 5 (8.5%) | 0 (0.0%) | 0 (0.0%) | 0 (0.0%) | 0 (0.0%) | 0 (0.0%) |  | 0 (0.0%) | 0 (0.0%) | 0 (0.0%) | 0 (0.0%) | 0 (0.0%) | 0 (0.0%) | 59 (100%) | |
| VERT | 448 (61.4%) | 0 (0.0%) | 81 (11.1%) | 121 (16.6%) | 1 (0.1%) | 0 (0.0%) | 0 (0.0%) | 0 (0.0%) | 0 (0.0%) |  | 0 (0.0%) | 1 (0.1%) | 4 (0.5%) | 74 (10.1%) | 5 (0.7%) | 79 (10.8%) | 730 (100%) | |
| CSW | 308 (37.2%) | 0 (0.0%) | 234 (28.3%) | 59 (7.1%) | 0 (0.0%) | 0 (0.0%) | 0 (0.0%) | 0 (0.0%) | 0 (0.0%) |  | 0 (0.0%) | 0 (0.0%) | 0 (0.0%) | 227 (27.4%) | 0 (0.0%) | 227 (27.4%) | 828 (100%) | |
| **Ethiopia** | | | | | | | | | | | | | | | | | |  |
| HET | 1 (0.4%) | 1 (0.4%) | 225 (97.8%) | 1 (0.4%) | 0 (0.0%) | 0 (0.0%) | 0 (0.0%) | 0 (0.0%) | 0 (0.0%) |  | 0 (0.0%) | 2 (0.9%) | 0 (0.0%) | 0 (0.0%) | 2 (0.9%) | 2 (0.9%) | 230 (100%) |  |
| **Central Africa** | | | | | | | | | | | | | | | | | | |
| HET | 761 (28.5%) | 15 (0.6%) | 251 (9.4%) | 179 (6.7%) | 103 (3.9%) | 180 (6.7%) | 152 (5.7%) | 39 (1.5%) | 10 (0.4%) |  | 43 (1.6%) | 209 (7.8%) | 286 (10.7%) | 445 (16.6%) | 538 (20.1%) | 983 (36.8%) | 2,673 (100%) | |
| VERT | 50 (26.9%) | 2 (1.1%) | 32 (17.2%) | 17 (9.1%) | 3 (1.6%) | 16 (8.6%) | 4 (2.2%) | 3 (1.6%) | 0 (0.0%) |  | 0 (0.0%) | 7 (3.8%) | 22 (11.8%) | 30 (16.1%) | 29 (15.6%) | 59 (31.7%) | 186 (100%) | |
| **Southern Africa** | | | | | | | | | | | | | | | | | |  |
| HET | 235 (3.0%) | 27 (0.3%) | 7,273 (93.9%) | 113 (1.5%) | 1 (0.0%) | 13 (0.2%) | 0 (0.0%) | 2 (0.0%) | 0 (0.0%) |  | 0 (0.0%) | 3 (0.0%) | 34 (0.4%) | 42 (0.5%) | 37 (0.5%) | 79 (1%) | 7,743 (100%) |  |
| MSM | 4 (1.5%) | 33 (12.6%) | 217 (83.1%) | 0 (0.0%) | 4 (1.5%) | 0 (0.0%) | 0 (0.0%) | 0 (0.0%) | 0 (0.0%) |  | 0 (0.0%) | 0 (0.0%) | 0 (0.0%) | 3 (1.1%) | 0 (0.0%) | 3 (1.1%) | 261 (100%) |  |
| VERT | 20 (1.4%) | 2 (0.1%) | 1,379 (97.9%) | 6 (0.4%) | 0 (0.0%) | 0 (0.0%) | 0 (0.0%) | 0 (0.0%) | 0 (0.0%) |  | 0 (0.0%) | 0 (0.0%) | 2 (0.1%) | 0 (0.0%) | 2 (0.1%) | 2 (0.1%) | 1,409 (100%) |  |

Regional distribution of HIV-1 subtypes, CRFs, and URFs within key populations in 1990-2015.

*Total CRFs is the sum of CRF01_AE, CRF02_AG, and Other CRFs. †Total Recombinants is the sum of total CRFs and URFs. ‡Total is the sum of total recombinants and all HIV-1 subtypes.

Abbreviations: BLOOD = Blood/plasma transfusion associated infections, CRF = Circulating Recombinant Form, CSW = Commercial sex workers, HET = Heterosexual, MSM = Men who have sex with men, PWID = People who inject drugs, URF = Unique Recombinant Form, VERT = Vertical transmission (mother to child).
